# Evaluation of the metabolic activity, angiogenic impacts, and GSK-3β signaling of the synthetic cannabinoid MMB-2201 on human cerebral microvascular endothelial cells

**DOI:** 10.1186/s42238-024-00255-7

**Published:** 2024-12-20

**Authors:** Laith Naser AL-Eitan, Saif Zuhair Alahmad, Sufyan Ali Ajeen, Ahmad Younis Altawil, Iliya Yacoub Khair, Hana Salah Abu Kharmah, Mansour Abdullah Alghamdi

**Affiliations:** 1 Department of Biotechnology and Genetic Engineering, Jordan University of Science and Technology, P.O. Box 3030, Irbid, 22110 Jordan; 2 Department of Anatomy, College of Medicine, King Khalid University, Abha, 62529 Saudi Arabia; 3 Genomics and Personalized Medicine Unit, The Centre for Medical and Health Research, King Khalid University, Abha, 62529 Saudi Arabia

**Keywords:** Angiogenesis, Cannabinoid receptors, GSK-3β, MMB-2201, VEGF, Synthetic cannabinoid

## Abstract

Angiogenesis is an intrinsic physiological process involving the formation of new capillaries from existing ones. Synthetic cannabinoids refer to a class of human-made chemicals that are primarily designed to mimic the effects of delta-9-tetrahydrocannabinol, the primary psychoactive compound in cannabis. Studies investigating the association between synthetic cannabinoids and cellular reactions are limited, and the available scientific evidence is insufficient. Consequently, the primary goal was to examine the effects of the synthetic cannabinoid MDMB-2201 on brain angiogenesis in vitro to provide a comprehensive analysis of MMB-2201’s potential therapeutic or adverse effects on vascular development and related health conditions. Human Cerebral Microvascular Endothelial Cells (HBEC-5i) were incubated with MMB-2201, and their metabolic activity, migration rate, and tubular structure formation were examined. Expression levels of several angiogenesis-related proteins such as vascular endothelial growth factor (VEGF), Angiopoietin-1 (ANG-1), and Angiopoietin-2 (ANG-2) were assessed using western blot, ELISA, and real-time PCR. Furthermore, the phosphorylation of glycogen synthase kinase 3 beta (GSK-3β) at Ser9 induced by MMB-2201 was evaluated. HBEC-5i cells showed a significant increase in metabolic rate, enhanced migration, and sprouting of brain endothelial cells. Moreover, there was a noticeable increase in the mRNA and protein levels of VEGF, ANG-1, and ANG-2, as well as in the phosphorylation rate of GSK-3β at Ser9. This study paves the way for a novel pharmacological approach to addressing various angiogenesis-related diseases by targeting cannabinoid receptor type-1. Further exploration using different antagonists or agonists of cannabinoid receptors, depending on the specific characteristics of the disorders, may be necessary.

**Supplementary Information:**

The online version contains supplementary material available at 10.1186/s42238-024-00255-7.

## Introduction

Angiogenesis is generally defined by the emergence of new vascular capillaries through continuous growth and expansion of pre-existing vascular structures (Adair and Montani 2010). This complex and highly regulated process involves several distinct steps (Adair and Montani 2010; Carmeliet and Jain 2011). Brain angiogenesis has been implicated in several cognitive functions, such as the learning and memory processes (Kerr et al. 2010). Several angiogenesis-related proteins, including Vascular Endothelial Growth Factor (VEGF), Fibroblast Growth Factor (FGF), Angiopoietin-1 (ANG-1), and − 2 (ANG-2), and others, stimulate intracellular signaling pathways in the endothelial cells, initiating angiogenesis. Consequently, endothelial cells can transform into tip endothelial cells that facilitate navigation through a series of processes to enhance cell migration (Carmeliet and Jain 2011). Many studies have provided substantial evidence supporting the efficacy of angiogenic-related proteins in reducing neurological impairments and facilitating stroke recovery through the promotion of angiogenesis (Zhang et al. 2000). The serine/threonine kinase Glycogen Synthase Kinase-3β (GSK-3β) has been identified and investigated for its involvement in glycogen synthesis, proliferation, and brain functions (Jaworski et al. 2019). It is also strongly associated with the regulation of brain plasticity, memory formation, and the behavioral patterns stimulated by drug addiction (Barr and Unterwald 2020; Marosi et al. 2022). Regulation of GSK-3β occurs through the phosphorylation of specific amino acid residues. The phosphorylation of Tyr216 activates it, whereas its inactivation is attributed to Ser9 phosphorylation (Barr and Unterwald 2020; Hur and Zhou 2010; Kim et al. 2002). GSK-3β has also been shown to be a critical regulator of angiogenesis, influencing migration and survival in endothelial cells and modifying vessel formation (Kim et al. 2002; Kobayashi et al. 2006). Furthermore, GSK-3β inhibition results in a reduction in HIF-1α degradation. Increased levels of HIF-1α stimulate VEGF secretion, which triggers the initiation of the Wnt/β-catenin signaling pathway and initiates angiogenesis (Holmes et al. 2008; Lee et al. 2009).

**Fig. 1:**
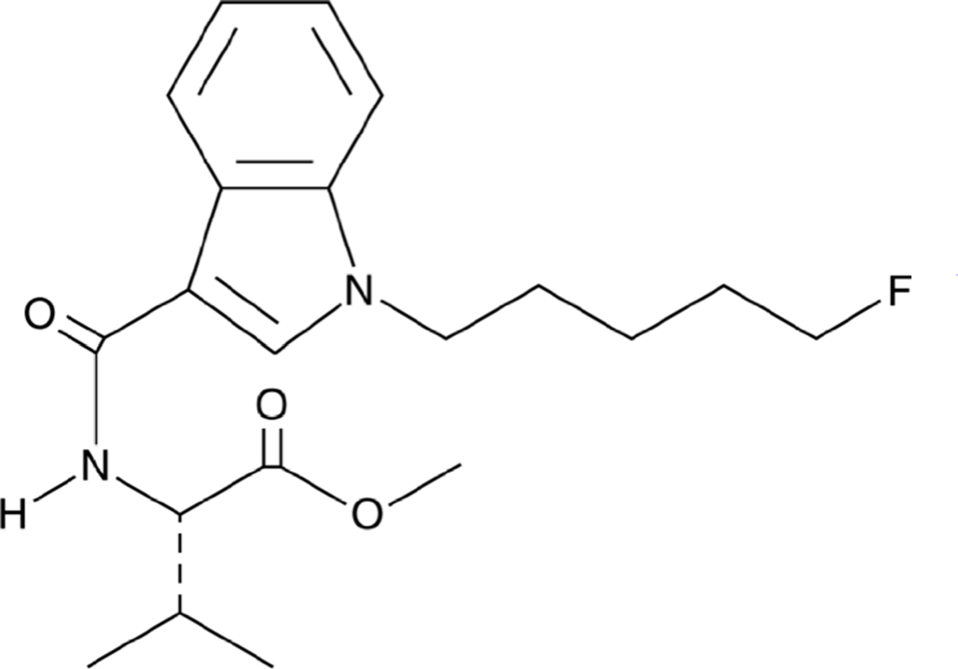
The chemical structure of the synthetic cannabinoid MMB-2201

Synthetic Cannabinoids (SCs) are recently established psychoactive chemicals designed to emulate the effects of natural cannabis in pharmaceutical investigations (Schurman et al. 2020; Tai and Fantegrossi 2014; Walsh and Andersen 2020). They contribute directly to identifying and characterizing the endocannabinoid system and its two receptor types: cannabinoid receptor type-1 (CBR-1), which is highly expressed in the brain, and type-2 (CBR-2), which is primarily distributed in immune system cells(Mackie 2008; Zou and Kumar 2018). However, misuse of SCs in some laboratories and institutions has led to their recreational use (Le Boisselier et al. 2017). SCs are highly addictive substances due to their elevated affinity for both cannabinoid receptors compared to THC (Cohen and Weinstein 2018). The synthetic cannabinoid “’MMB–2201"’ N-(1-(5-fluorophenyl)--1 H-indol–3-yl) carbonyl)--L-valine, methyl ester, also referred to as I-AMB, 5-fluoro MMB-PICA, and 5-fluoro AMB-PICA, is classified as a potent compound with an indole-3-carboxamide structure (Fig. 1 (C_20_H_27_FN_2_O_3_). The U.S. Drug Enforcement Administration (DEA) first documented MMB-2201 in drug seizures in 2018 (Yin 2019). MMB-2201 is an analog of the known CBR-1 agonist 5 F-AMB (Shevyrin et al. 2015). MMB-2201 has been restricted in several countries due to its high potency in causing addiction without clear medical justification (Barceló et al. 2017; Gaunitz 2022). The potential effects of MMB-2201 on brain angiogenesis remain poorly understood, particularly regarding endothelial cell viability, migration, and tube formation. Given the critical role of angiogenesis in both physiological and pathological contexts, this study aims to address this gap to examine the impact of MMB-2201 on brain angiogenesis. Moreover, this research seeks to elucidate the molecular mechanisms underlying MMB-2201’s effects on key steps in angiogenesis, including endothelial cell viability, migration, and tube formation. By focusing on these aspects, the study aims to comprehensively analyze MMB-2201’s potential therapeutic or adverse effects on vascular development and related health conditions by focusing on these aspects.

## Materials and methods

### Cell line and MMB-2201 Preparation

Human cerebral microvascular endothelial cells (HBEC-5i) (CRL–3245) were obtained by ATCC (ATCC; Manassas, VA). These cells were cultured in DMEM/F12 media (Euroclone S.p.A, Pero, Italy) supplemented with Microvascular Endothelial Cell Growth Kit-BBE (PCS-110–040; ATCC, Manassas, USA). This growth kit included heparin sulfate, bovine brain extract, hydrocortisone, ascorbic acid, rh-EGF, L-glutamine, 10% fetal bovine serum, and 1% penicillin and streptomycin. The cells were cultured in a 37 °C environment with a 5% CO_2_ concentration. Cell passaging was performed at 1:4 when the cells reached 80% confluence.

The synthetic cannabinoid MMB-2201 was purchased from Cayman Chemical Company (Ann Arbor, Michigan, USA). Dimethyl sulfoxide (DMSO) was used to prepare MMB-2201 stock solution with a concentration of 2 mg/mL. Consistent DMSO concentrations were maintained throughout the study. Subsequently, serial dilution was performed to create six distinct concentrations from the stock solution, with final concentrations of (0.0001, 0.001, 0.01, 0.1, and 1 µM. The control group was prepared using serum-free media supplemented with DMSO, which served as a baseline for evaluating the effects of MMB-2201 on HBEC-5i. The final concentration of DMSO in the control group was 0.1%.

### Assessing cellular metabolic activity

The metabolic rate of HBEC-5i cells was assessed using the MTT colorimetric assay which measures enzymatic activity leading to the formation of a purple formazan product. Approximately 5000 HBEC-5i cells were seeded per well in a 96-well plate and incubated for 24 h. Subsequently, the cells were treated with MMB-2201 at various concentrations (0.0001–1 µM), alongside a control group. Following treatment, the HBEC-5i cells were then to MTT stock solution (5 mg/ml), with 10 µl added to each well containing 100 µL of culture media. The plate was incubated for 4 h. Formazan crystals were solubilized by adding DMSO, followed by 15 minutes of agitation. Absorbance was measured at 570 nm using an ELISA reader. Each concentration was tested in triplicate.

### Endothelial cell migration assay

The ability of endothelial cell to migrate following treatment with MMB-220 was evaluated using an Endothelial cell migration assay. Briefly, HBEC-5i cells were seeded in a 12-well plate and grown until they reached a confluence of 90–100%. A wound approximately 1 mm wide was created in the monolayer using a 1,000 µL pipette tip. The detached cells resulting from the scratching procedure were carefully washed away with PBS. Subsequently, the cells were treated with a range of MMB-2201 concentrations (0.0001–1 µM) and a control group for 24 hours. Four images of the wound area were captured for each well at baseline and 24 h after treatment using a camera mounted on an inverted microscope to monitor the wound healing process. ImageJ software was used to measure the wound area to quantify the migration at the baseline (0 h) and after 24 h. The percentage reduction in wound area was determined by subtracting the total wound distance from the average exposure distance, dividing it by the total wound area, and multiplying by 100%. Each concentration was tested in triplicate.

### Capillary tube formation assay

The angiogenic capacity induced by MMB-2201 in HBEC-5i cells was investigated using a Matrigel capillary tube formation assay. A total of 2 × 10^4^ HBEC-5i) cells were seeded per well in a 96-well plate. The cells were then treated with MMB-2201 in varying concentrations (0.0001, 0.01, and 1 µM) for 24 h. The formation of tube-like structures by endothelial cells was assessed using microscopic images captured after 24 hours post-treatment. The number of tubes, branching points, loops, and total tube length were measured to quantify the angiogenic response.

### Immunoblotting

Immunoblotting was employed to quantify the phosphorylation of GSK-3β at Ser9 and the protein expression levels of VEGF, ANG-1, and ANG-2 in HBEC-5i. HBEC-5i cells were treated with varying concentrations of MMB-2201 for 24 h. Cell lysis was performed using RIPA buffer mixed with mini tablets of protease-phosphatase inhibitors.Protein quantification was carried out using a protein assay kit (Bio-Rad, Hercules, CA, USA), following the manufacturer’s instructions. Approximately 20 µg of protein was loaded onto SDS-PAGE gel and subsequently transferred to a PVDF membrane. The membrane was incubated overnight at 4 °C with primary antibodies listed in Supplementary Table 1. Afterward, HRP-conjugated secondary antibodies were applied for 2 hours. Densitometric analysis of the blots was performed using ImageJ software by measuring the area of individual bands.

### Enzyme-linked immunosorbent assay (ELISA)

Furthermore, the concentrations of VEGF, ANG-1, and ANG-2 released into the culture media were measured using ELISA. The cultured media were collected and centrifuged (10,000 rpm/10 minutes) after treating the HBEC-5i cells with different concentrations of MMB-2201 for 24 h. ELISA kits for VEGF (ab100662), ANG-1 (ab99972), and ANG-2 (ab99971) were obtained from Abcam (Cambridge, MA) and used following the manufacturer’s recommendations. The cultured media were diluted 5-fold before performing the ELISA assays.

### Quantitative real-time PCR (qRT-PCR)

Total RNA was extracted from HBEC-5i cells, and the purity and concentration of RNA were evaluated using a Nanodrop ND-1000 device (Bio Drop, UK). All samples exhibited acceptable levels of purified RNA. Reverse transcription and amplification were performed using SOLIScript® 1-step SolisGreen® Kit (08-63-00250; Solis BioDyne). Reverse-transcribed DNA amplification was conducted under the conditions represented in Supplementary Table 2. The expression levels of three genes (VEGF, ANG-1, and ANG-2) were measured. The β-actin gene was used as a reference gene. The primers used in this method are listed in Supplementary Table 3.

### Statistical analysis

The statistical analysis was conducted utilizing GraphPad Prism (version 9.0.0). The statistical differences between multiple groups were evaluated using a one-way ANOVA followed by a post-hoc Tukey test. All data are presented as the mean ± standard error of means (L. N. Al-Eitan et al. 2023). Differences were considered statistically significant at *p* < 0.05. The chemical structure (Fig. 1) was drawn using the data provided by the supplier of the compound, ensuring accuracy in representing the structure of MMB-2201.

## Results

### The regulation of cannabinoid receptor type 1 (CB1-R) expression plays a critical role in the angiogenesis process 

**Fig. 2:**
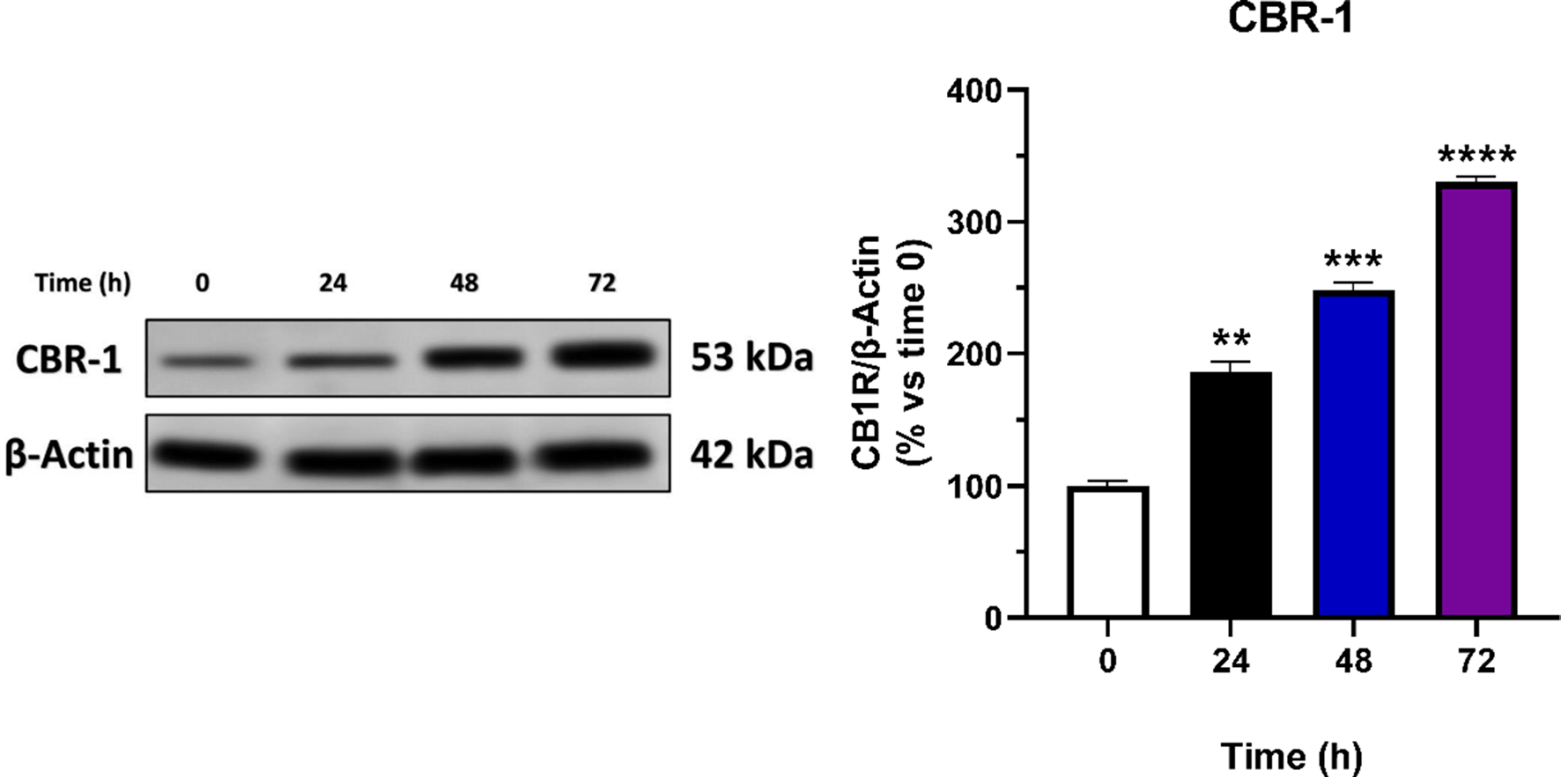
Cannabinoid receptor type 1 (CBR-1) has been shown to increase during the stimulation of the angiogenesis process. HBEC-5i cells were cultured in proangiogenic media supplemented with bFGF (10 ng/mL) for 24, 48, and 72 h. (**A**) Western blot images were obtained, and densitometric analysis of the CBR-1 bands was conducted using ImageJ software. (**B**) Quantitative analysis of the CBR-1 expression rates. Data are presented as mean ± SEM (*n* = 3). (**) indicates *p* < 0.01. (***) indicates *p* < 0.001. (****) indicates *p* < 0.0001

The investigation began by studying quiescent HBEC-5i cells at time 0 to examine the changes of CBR-1 expression during angiogenesis. The cells were then treated with proangiogenic media supplemented with bFGF (10 ng/mL) to induce proliferation. CBR-1 protein levels were assessed at 24, 48, and 72 h. Remarkably, the protein expression of the CBR-1 receptor increased gradually across time, with significant differences observed at 24 h (*p = 0.001*), 48 h (*p = 0.0001*), and 72 h (*p < 0.0001*) (Fig. 2). These findings suggest an association between the CBR-1 receptor and angiogenesis.

### MMB-2201 enhanced the metabolic activity of brain endothelial cells

**Fig. 3:**
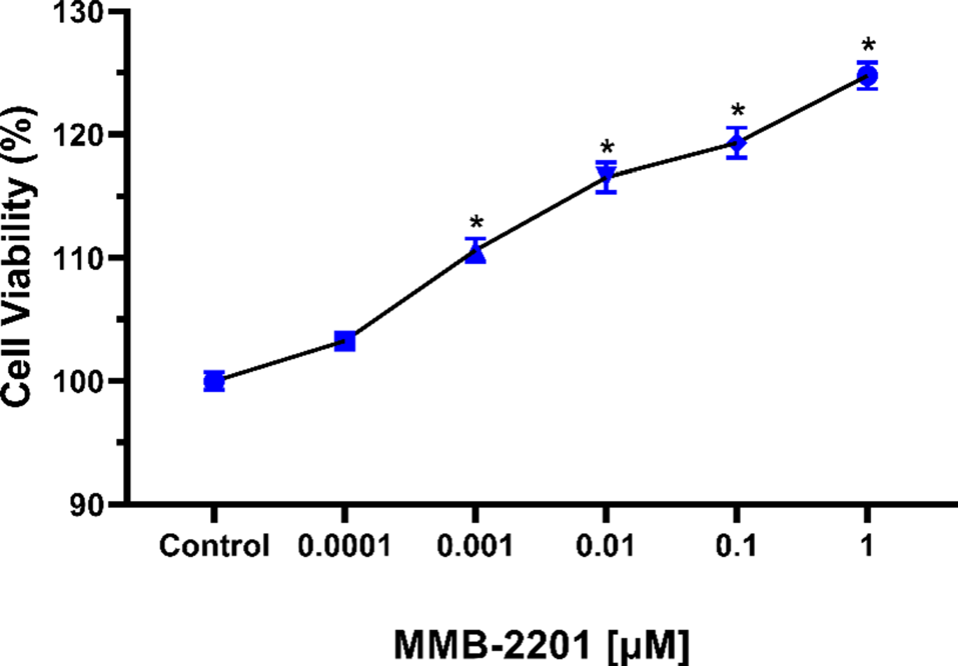
The synthetic cannabinoid MMB-2201 increased the metabolic activity in endothelial cells. A total of 5000 HBEC-5i cells were counted and seeded in a 96-well plate for 24 h. Different concentrations of MMB-2201 were then incubated with the cells for 24 h. Subsequently, the media containing MMB-2201 was discarded, and the cells were treated with MTT solution and incubated for 4 h at 37 °C and 5% CO_2_. The formazan crystals were then solubilized with DMSO, and absorbance was measured. The treatment significantly enhanced cell viability at concentrations ranging from 0.001 µM to 1 µM compared to the control. Data are presented as mean ± SEM (*n* = 3). (*) indicates *p* < 0.05

The MTT assay was conducted to assess the impact of MMB-2201 on the HBEC-5i cells. The metabolic activity of HBEC-5i was significantly enhanced in MMB-2201-treated cells, with an increase observed at 0.001 µM (*p = 0.0001*), 0.01 µM, 0.1 µM, and 1 µM MMB-2201(*p < 0.0001*). No significant difference was found at 0.0001 µM MMB-2201 (*p = 0.1242*) compared to the control (Fig. 3). These findings suggest that MMB-2201 enhances the proliferation rate without inducing toxicity, potentially reducing the apoptosis rate of HBEC-5i cells.

### MMB-2201 promoted the migration of endothelial cells in *vitro*

**Fig. 4:**
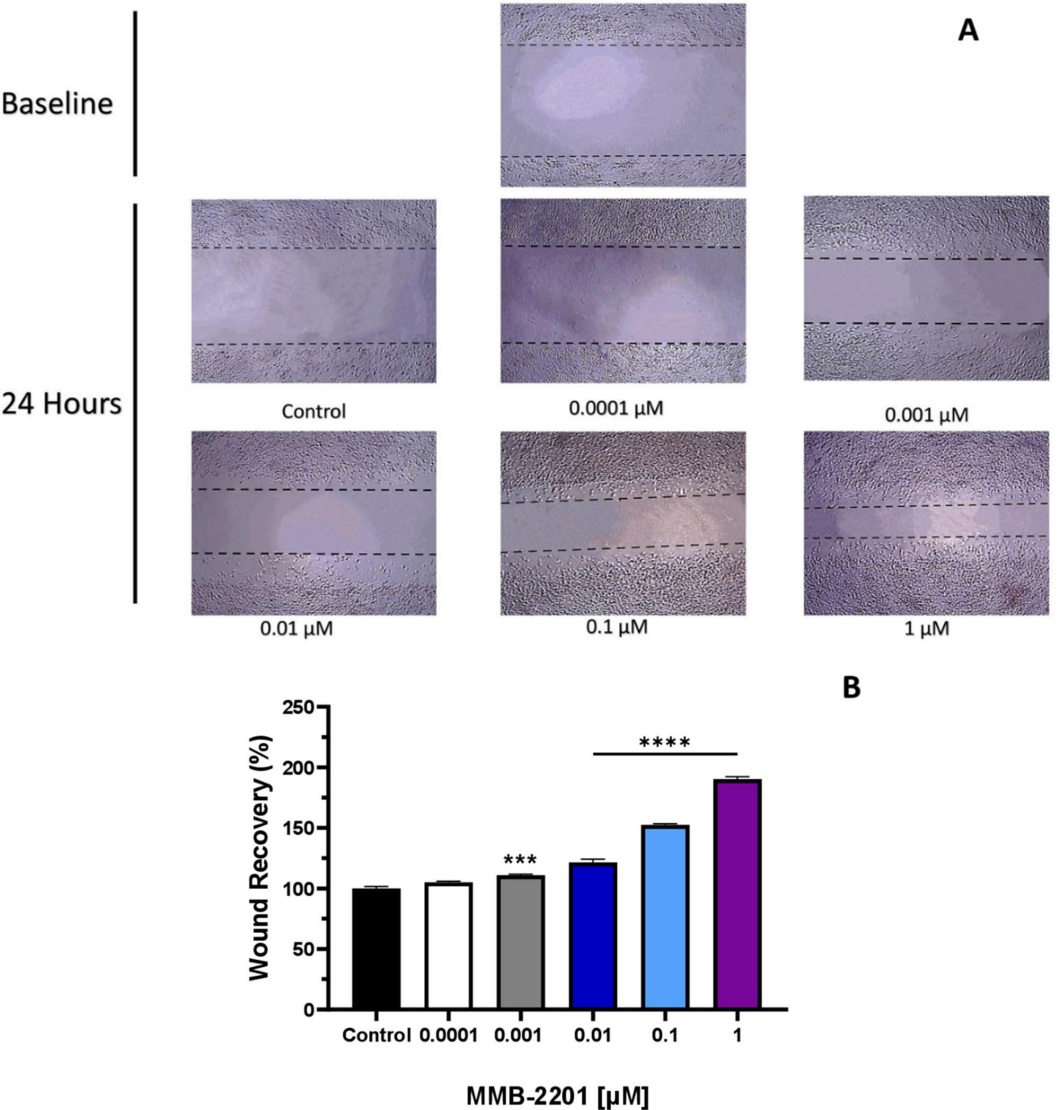
The synthetic cannabinoid MMB-2201 promoted the migration rate of endothelial cells. A wound was introduced in the confluent endothelial monolayer in 12-well plates. Different concentrations of MMB-2201 were incubated with the cells for 24 h. (**A**) Microscopic images of migrated HBEC-5i cells were captured at baseline (Zero time) and after 24 h. (**B**) Quantitative analysis of the migration data revealed a significant increase in cell migration in cells treated with different concentrations of MMB-2201. Data are presented as mean ± SEM (*n* = 3). (***) indicates *p* < 0.001. (****) indicates *p* < 0.0001

To investigate the potential of MMB-2201 in promoting endothelial cell migration, we conducted an endothelial cell migration assay. HBEC-5i cells were exposed to different concentrations of MMB-2201(0.0001 to 1 µM) after creating a scratch in the confluent monolayer of endothelial cells (Fig. 4A**).** The treated groups showed a significant increase in migration rate compared to the untreated control group. Endothelial cell migration was significantly enhanced at concentrations of 0.001 µM (*p = 0.0004*), 0.01 µM, 0.1 µM, and 1 µM (*p < 0.0001*) of MMB-2201 (Fig. 4B). These findings suggest that MMB-2201 promotes the migration of HBEC-5i cells, a critical step in angiogenesis.

### MMB-2201 has induced the angiogenic capacity of HBEC-5i cells

**Fig. 5:**
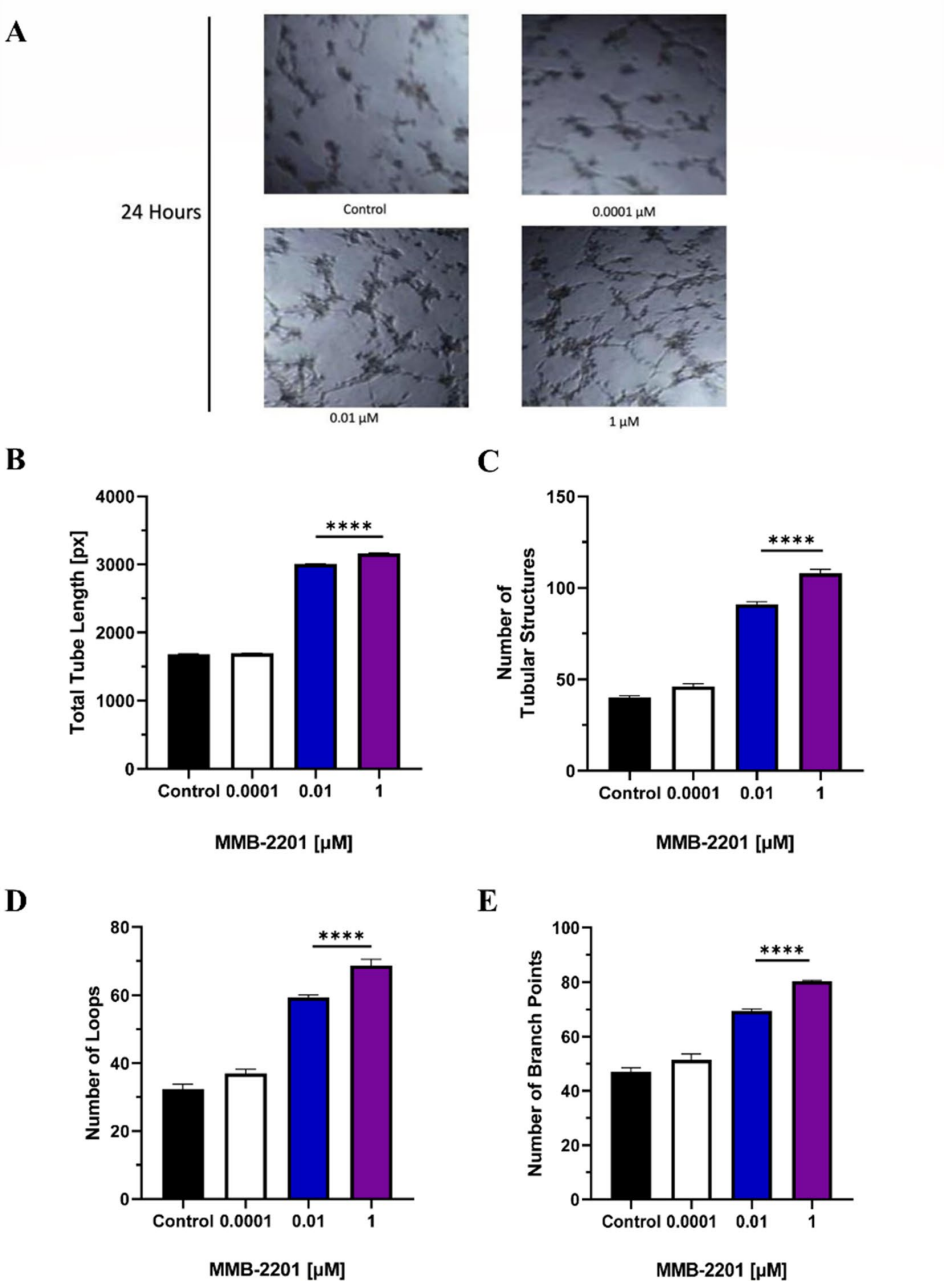
The formation of angiogenic tubular structures was induced in endothelial cells after treatment with the synthetic cannabinoid MMB-2201. A total of 20,000 HBEC-5i cells were seeded in a pre-cooled 96-well plate coated with basement membrane extract. (**A**) Microscopic images captured 24 h after treatment with MMB-2201 illustrate the development of tubular structures. A quantitative analysis of the angiogenic parameters was conducted, including (**B**) total tube length, (**C**) the number of tubular structures, (**D**) the number of loops, and (**E**) the number of branches. A significant increase was observed at concentrations of 0.01 µM and 1 µM of MMB-2201. Data are presented as mean ± SEM (*n* = 3). (****) indicates *p* < 0.0001

Since sprouting brain endothelial cells is crucial for brain angiogenesis, we further examined the effects of MMB-2201 on HBEC-5i cells in vitro. A matrigel endothelial cell tube formation assay was used to assess whether MMB-2201 could promote brain endothelial cell angiogenesis. Four Angiogenic parameters were investigated: the number of tubular structures, the number of branches, the number of loops, and the total tube length. HBEC-5i cells were incubated with three concentrations of MMB-2201 (0.0001 µM, 0.01 µM, and 1 µM) (Fig. 5A). As expected, significant differences were observed in all angiogenic parameters at 0.01 µM and 1 µM. These results indicate that MMB-2201 could promote brain angiogenesis (Fig. 5B -E).

### The expression of several angiogenesis-associated genes was upregulated following treatment with MMB-2201

**Fig. 6:**
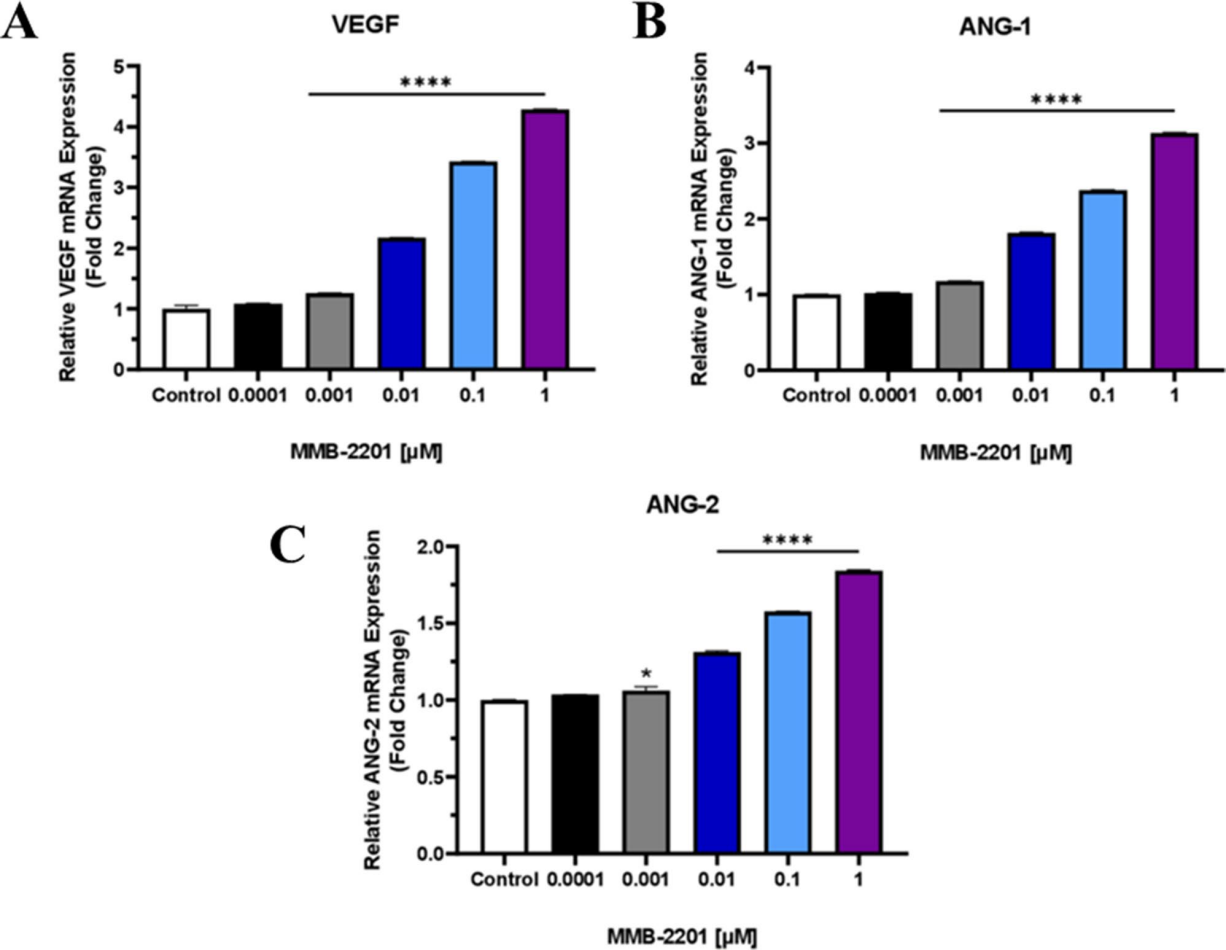
Effects of the synthetic cannabinoid MMB-2201 treatment on mRNA expression in endothelial cells. The results of qRT-PCR showed the upregulation of several angiogenic genes, including VEGF, ANG-1, and ANG-2, at different concentrations of MMB-2201. Data are presented as mean ± SEM (*n* = 3). (*) indicates *p* < 0.05. (****) indicates *p* < 0.0001

After confirming that MMB-2201 enhances endothelial cell migration and sprouting, promoting brain angiogenesis, we investigated its effects on the expression of angiogenesis-related genes. The expression levels were evaluated using RT-qPCR, ELISA, and immunoblotting. RT-qPCR analysis showed a significant upregulation of VEGF mRNA in MMB-2201-treated cells, with an increases of 1.3-fold at 0.001 µM, 2.2-fold at 0.01 µM, 3.4-fold at 0.1 µM, and 4.3-fold at 1 µM (*p < 0.0001*). Similarly, ANG-1 mRNA was also upregulated, showing increases of 1.2-fold at 0.001 µM, 1.8-fold at 0.01 µM, 2.4-fold at 0.1 µM, and 3.1-fold at 1 µM (*p < 0.0001*) in HBEC-5i cells. Additionally, there was an increase in ANG-2 mRNA expression following MMB-2201 treatment, with increases of 1.3-fold at 0.01 µM, 1.6-fold at 0.1 µM, and 1.8-fold at 1 µM (*p < 0.0001*) (Fig. 6).

**Fig. 7:**
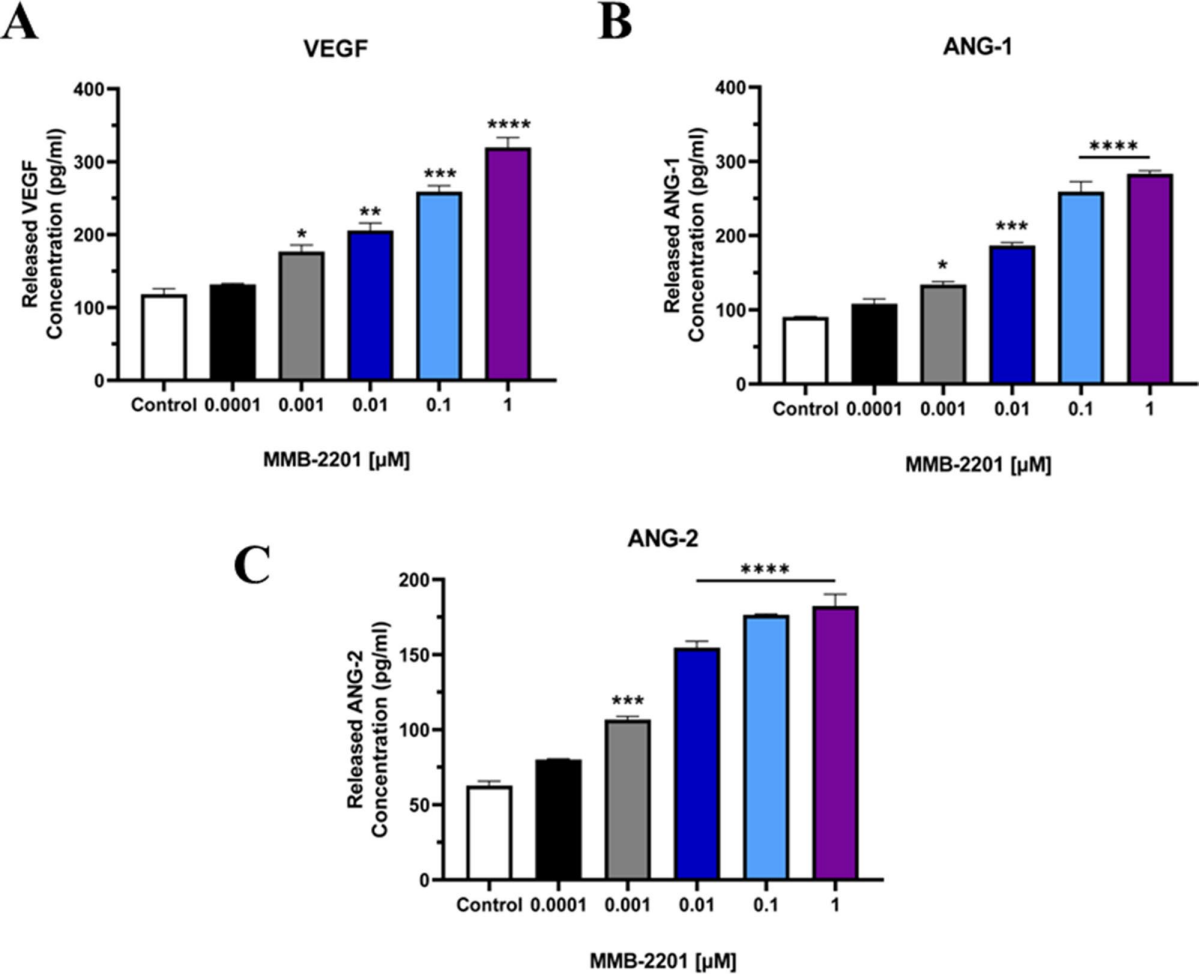
The synthetic cannabinoid MMB-2201 increased the release of VEGF, ANG-1, and ANG-2 from endothelial cells. VEGF, ANG-1, and ANG-2 release from HBEC-5i was investigated using ELISA. The release of these proteins was significantly increased at different concentrations of MMB-2201. Data are presented as mean ± SEM (*n* = 3). (*) indicates *p* < 0.05. (**) indicates *p* < 0.01. (***) indicates *p* < 0.001. (****) indicates *p* < 0.0001

**Fig. 8:**
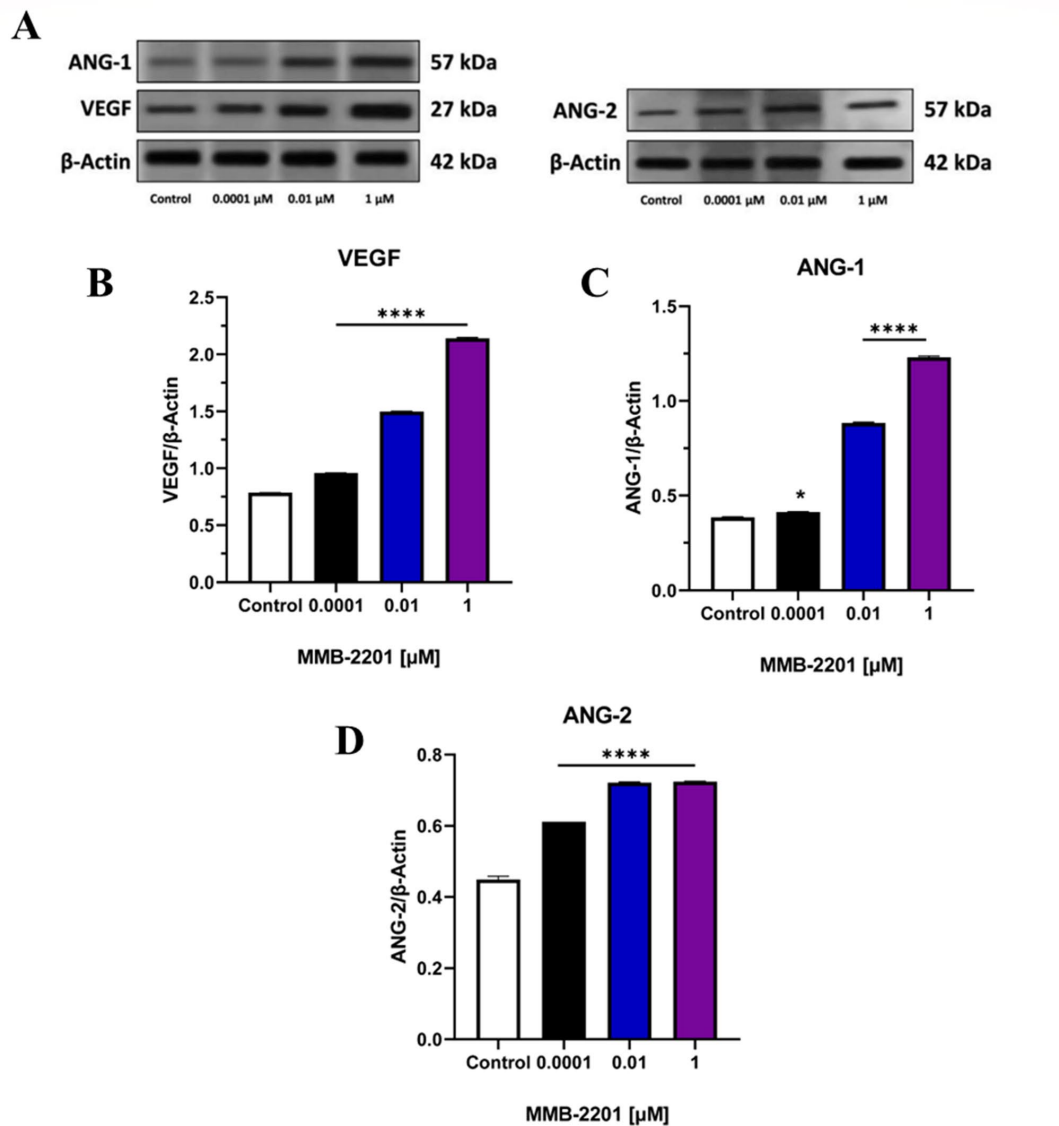
The synthetic cannabinoid MMB-2201 elevated the intracellular expression of VEGF, ANG-1, and ANG-2 in endothelial cells. The protein levels were analyzed using western blot analysis and specific primary antibodies. (**A**) Representative blot showing the results. Quantification of the expression levels of (**B**) VEGF, (**C**) ANG-1, and (**D**) ANG-2. Data are presented as mean ± SEM (*n* = 3). (*) indicates *p* < 0.05. (****) indicates *p* < 0.0001

In addition, the secretion levels of VEGF, ANG-1, and ANG-2 into the media were investigated using ELISA. Stimulation VEGF, ANG-1, and ANG-2 secretion by HBEC-5i cells was observed following treatment with MMB-2201 (Fig. 7). Immunoblotting analysis was also performed to assess the intracellular expression of VEGF, ANG-1, and ANG-2. Specific bands corresponding to VEGF at approximately 27 kD were detected in MMB-2201-treated HBEC-5i cells and the control group (Fig. 8A). Significant upregulation of VEGF protein expression was observed at concentrations of 0.0001 µM, 0.01 µM, and 1 µM of MMB-2201(*p < 0.0001*) (Fig. 8B). Similarly, bands corresponding to ANG-1 and ANG-2 proteins at approximately 57 kD were detected in HBEC-5i cells treated with various concentrations of MMB-2201. Treatment with MMB-2201 resulted in a significant upregulation of both ANG-1 and ANG-2 expression (Fig. 8C, D).

### The phosphorylation level of GSK-3β at the Ser9 residue increased following MMB-2201 treatment

**Fig. 9:**
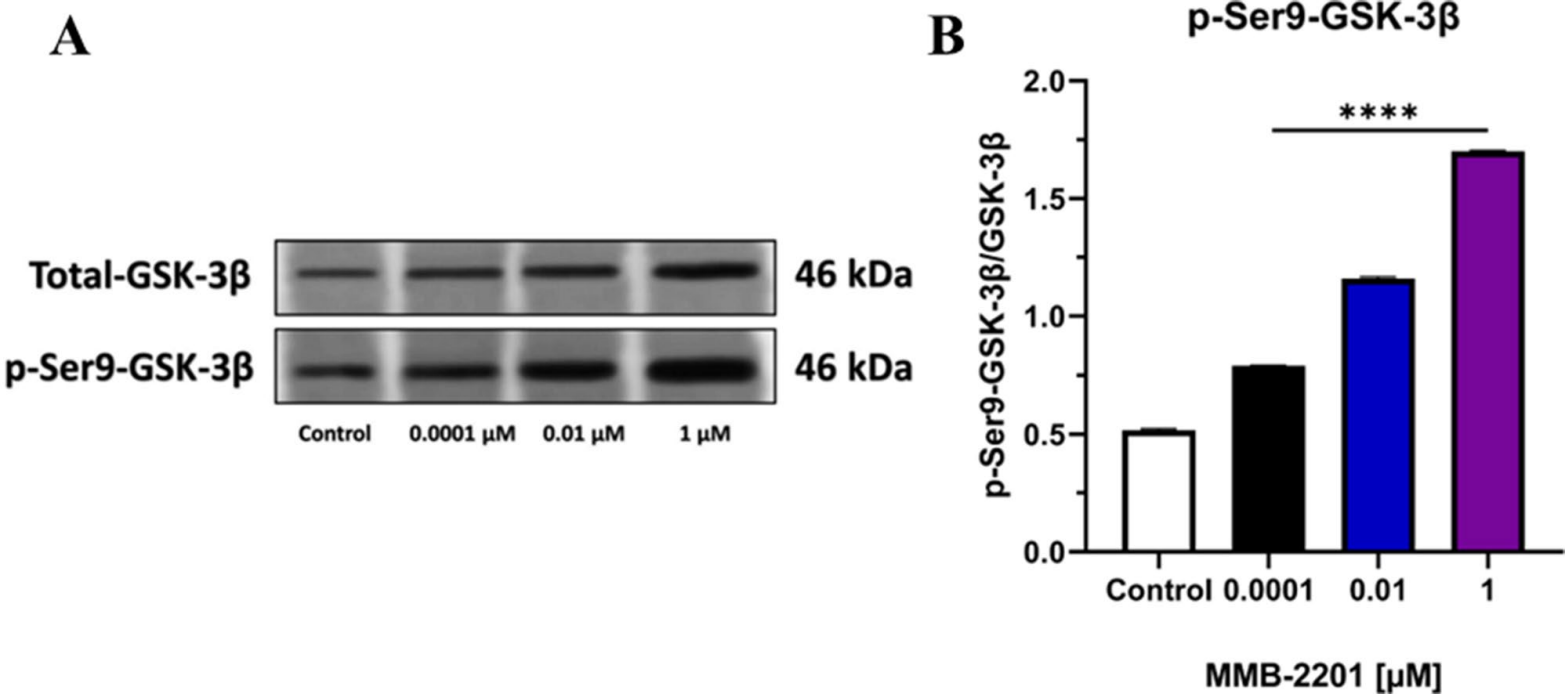
The synthetic cannabinoid MMB-2201 enhanced the phosphorylation of GSK-3β at the Ser9 residue in endothelial cells. The phosphorylation levels were analyzed using western blot analysis and specific primary antibodies. (**A**) A representative blot showing the results. (**B**) Quantification of the expression and phosphorylation levels of p-Ser9-GSK-3β. The data are presented as mean ± SEM (*n* = 3). (****) indicates *p* < 0.0001

We examined whether MMB-2201 increases (the phosphorylation of GSK-3β at the Ser9 residue). Bands representing total and phosphorylated GSK-3β were observed at approximately 46 kD in cells treated with MMB-2201 (Fig. 9A). In summary, there was a significant enhancement in GSK-3β phosphorylation at the Ser9 residue relative to the total GSK-3β levels (Fig. 9B).

## Discussion

Many studies have highlighted the significance of endocannabinoid system receptors as therapeutic targets for metastatic and angiogenic-related conditions and their role in controlling disease development and progression (Hinz 2022; Hohmann et al. 2019; Glogauer 2022). The mechanism of these endocannabinoid-targeting drugs varies based on the type of cancer and the receptor, CBR-1, CBR-2, transient receptor potential vanilloid TRPV1 or TRPV2, and whether it is dependent or independent. Previous studies have reported that blocking CBR-1 signaling inhibits cancer growth in mantle cell lymphoma, thyroid, colon, and breast cancers in vitro and in vivo (Portella et al. 2003; Flygare 2005; Sarnataro et al. 2006; Santoro 2009; Luo 2019; Faiz et al. 2024). Anti-angiogenesis therapies have recently been successfully introduced into the clinic to treat common angiogenesis-related diseases (Carmeliet 2005; Ferrara 2005; Annex 2021; Moriya 2017; Battaglin et al. 2018). Interestingly, these drugs have been found to effectively inhibit tumor angiogenesis in vivo by acting on both CBR-1 and CBR-2, thereby inhibiting cancer growth, particularly by suppressing VEGF signaling via inducing endothelial cell apoptosis (Casanova, 2003; Blázquez, 2003; Portella 2003; Pisanti 2009; Grimaldi 2006; Sheik et al. 2023; Prateeksha et al. 2023; Faiz et al. 2024; Wang et al. 2019).

Synthetic cannabinoids (SCs) have been designed to mimic the function of natural endocannabinoid system receptor agonists such as anandamide and 2-arachidonoylglycerol (2-AG) (Justinová et al. 2011). Subsequent investigations into these newly established chemicals have demonstrated their potential to interfere with disease treatment (Bogdanović et al. 2017; Kokona et al. 2016). MMB-2201 is categorized under the indole-based family of synthetic cannabinoids whose chemical structure consists of an indole core with a substituted aminoalkylindazole group. The restrictions put on MMB-2201 in several countries are due to its high potency to induce addiction with no apparent medical justification (Barceló et al. 2017; Gaunitz 2022). Excessive consumption and long-term utilization of these substances can result in adverse effects, such as changes in mental state, loss of consciousness, and tachycardia (Barceló et al. 2017).

CBR-1 activation triggers a complex cascade of intracellular signaling pathways, significantly influencing various physiological processes. CBR-1, a G-protein-coupled receptor (GPCR), undergoes a conformational change upon ligand binding, activating associated Gi/o proteins (Tuteja 2009). This activation usually leads to the inhibition of adenylyl cyclase, thereby decreasing cyclic AMP (cAMP) levels within the cell (Howlett et al. 2010). The reduction in cAMP levels affects several signaling pathways. One major pathway affected is the(MAPK/ERK)pathway, which is crucial for cell proliferation and survival (Dillon et al. 2021). In endothelial cells, the activation of this pathway enhances their proliferation and migration, both of which are essential steps in angiogenesis (Pisanti and Bifulco 2009).

Additionally, CBR-1 activation stimulates the PI3K/AKT pathway (Howlett et al. 2010). While CBR-1 generally supports angiogenesis, it can also contribute to pathological conditions like cancer, where excessive angiogenesis can support tumor growth and metastasis. Thus, the activation of CBR-1 and its downstream effects are critical for both physiological and pathological angiogenesis, highlighting its complex role in vascular biology (Dudley and Griffioen 2023). A recent study involving in vitro and in vivo experiments demonstrated that neuronal cannabinoid CBR-1 receptors inhibit glutamatergic signaling, thereby suppressing the growth of melanoma brain metastases (Costas-Insua et al. 2023). Martinez-Martinez et al. analyzed the effect of CBR-2 receptor activation after treatment of the colonic epithelial tumor cell line HT29 with the CBR-2 agonist JWH-133. They found that CBR-2 was overexpressed and that activation correlated with increased metastasis and disease progression, suggesting that CBR-2 activation could be considered an antitumor therapy (Martínez-Martínez et al. 2015).

In recent decades, several investigations have examined the effects of SCs on various cultured human cell lines. Jiang et al. (2005) investigated the induction of proliferation and migration of neural stem cells, both in vivo and *in vitro and* they demonstrated an association between cannabinoid receptors and the regulation of neurogenesis (Jiang et al. 2005). On the other hand, the synthetic cannabinoid CB83 significantly reduced cell viability of the HT-29 colorectal adenocarcinoma cell line, underscoring the potential contribution of synthetic cannabinoids to the cancer treatment (Cerretani et al. 2020). At the same time, some researchers emphasize the future therapeutic potential of synthetic cannabinoids; not all drugs exhibit significant effects. For instance, JWH-018, a synthetic cannabinoid, was tested on the human neuronal cell line SH-SY5Y, but no significant effect on cell viability was observed (Sezer et al. 2020).

The migration of endothelial cells plays a critical role in both angiogenesis and cancer cell metastasis (Justus et al. 2014). A study by Zhang et al. found that THC significantly inhibited HEC-1B and An3ca cell proliferation and migration (Zhang et al. 2018). Brain angiogenesis is a tightly regulated process linked with neurogenesis and vascular recruitment (Palmer et al. 2000). Enhancing brain angiogenesis holds potential benefits for mitigating damage caused by neurological such as Parkinson’s disease and for improving recovery from ischemic stroke injuries (Xue et al. 2021; Yuan et al. 2013). The activation of cannabinoid receptors has been demonstrated to enhance angiogenesis by promoting migration, proliferation, and the formation of tubular structures in endothelial cells. Conversely, studies have reported that the knockdown or inhibition of cannabinoid receptors using antagonists impairs angiogenesis (Pisanti et al. 2011).

A previous study investigated the effect of CBR-1 receptor silencing using lentiviral shRNA vectors on cell cycle progression, proliferation, migration, and mitotic signal activation in human melanoma cells. Cells transduced with CBR-1 lentiviral shRNAs exhibited a significant reduction in viability, colony-forming capability, and cell migration compared to control cells (Carpi et al. 2017). Several indazole-based synthetic chemicals, including 5 F-MDMB-PICA, (R)-5 F ADB, and EMB-FUBINACA, have demonstrated increased proliferative and angiogenic capacities in vitro (Al-Eitan 2023; Al-Eitan 2024; Al-Eitan 2024; AL-Eitan 2023; AL-Eitan 2023; Al-Eitan 2024). Additionally, the synthetic cannabinoid XLR-11, which acts as an agonist of cannabinoid receptors, has also exhibited angiogenic effects in HBEC-5i. This includes enhanced migration, the formation of tube-like structures, and increased cell viability (Al-Eitan et al. 2020). However, some synthetic cannabinoids have shown anti-angiogenic properties. For instance, WIN 55,212-2, an agonist of cannabinoid receptors was found to decrease vascular formation and proliferation of human endometriotic cells through MAPK/Akt-mediated apoptotic signaling (Lingegowda et al. 2021). Another study used the MTT and invasion assay to assess the inhibitory effect of WIN 55,212-2 and selective CBR-1 receptor antagonist AM251 on K562 cells, a chronic myelogenous leukemia (CML) model. The study revealed that WIN 55,212-2 effectively reduced cell proliferation, while AM251 exhibited less efficacy (Gholizadeh et al. 2019). Similarly, URB447 has demonstrated anti-metastatic and anti-cancer effects in colon cancer and melanoma (Benedicto et al. 2022). Against this backdrop, we investigated the influence of MMB-2201 on both endothelial cell migration rates and angiogenic capacity in HBEC-5i.

The current study investigated three important angiogenic regulators: VEGF, ANG-1, and ANG-2. VEGF signaling, through the activation of vascular endothelial growth factor receptors (VEGFRs), promotes various vascular activities, including the expansion and maturation of the blood vessel lumen and the proliferation of the endothelial cell. Furthermore, VEGF is a crucial factor in promoting and regulating the migration of endothelial cells (Melincovici et al. 2018; Takahashi 2011). Anti-VEGF drugs have emerged as cancer therapy targets due to their ability to inhibit VEGFR signaling. Cannabinoid receptor activation has been hypothesized to significantly decrease VEGF levels in various cancer cell lines (Blázquez et al. 2003). ANG-1 and ANG-2 are members of the Angiopoietin family and bind to the tyrosine kinase Tie2 receptor (Melincovici et al. 2018). While ANG-1 predominantly stimulates the formation and stabilization of blood vessels, ANG-2 antagonizes these actions and can impair angiogenesis (Brindle et al. 2006). ANG-2 can exert a proangiogenic effect by stimulating endothelial cell proliferation, migration, and new vascular branch formation through sprouting in the presence of VEGF (Moritz et al. 2017). XLR-11 and various indazole-based synthetic chemicals have shown altered angiogenic behaviors of HBEC-5i cells, influencing the release of VEGF, ANG-1, and ANG-2. Moreover, elevated phosphorylation rates of GSK-3β indicate its role in blood vessel formation(AL-Eitan and Abu Kharmah 2023; Al-Eitan and Abusirdaneh 2024; Al-Eitan and Alahmad 2023; Al-Eitan and Alkhawaldeh 2023; AL-Eitan et al. 2023, 2024). In the current study, we revealed that MMB-2201 significantly increased the production of VEGF, ANG-1, and ANG-2 in the HBEC-5i cells following cannabinoid receptor activation. Our results suggest that VEGF, ANG-1, and ANG-2 may significantly promote angiogenesis in the brain induced by cannabinoid receptor activation.

Glycogen synthase kinase-3β (GSK-3β) involves several biological processes, such as angiogenesis, proliferation, and brain functions. Its activity is regulated through phosphorylation at specific residues. Phosphorylation at Ser9 keeps GSK-3β constitutively inactive, while activation occurs through phosphorylation at Tyr216 (Barr and Unterwald 2020; Kim et al. 2002). For example, the synthetic cannabinoid HU-210 has been demonstrated to enhance cellular proliferation rates in cerebellar granule cell precursors by activating CBR-1, which induces the phosphorylation of AKT and Ser9-p-GSK-3β, thus demonstrating the involvement of these signaling pathways in regulating granule cell proliferation (Trazzi et al. 2010). Similarly, the CBR-1 agonist Arachidonyl-2’-chloroethylamide (ACEA) has been reported to reduce cerebral ischemic damage through GSK-3β signaling. ACEA treatment promotes the phosphorylation of GSK-3β, enhancing mitochondrial biogenesis and providing a protective response to brain ischemia (Bai et al. 2017). GSK-3β signaling is also proposed as a potential therapeutic target for regulating angiogenesis in human glioma cells (Zhao et al. 2015). Suppression of GSK-3β activity via Ser9 phosphorylation may stimulate endothelial cells to produce proangiogenic proteins, thereby promoting angiogenesis (Holmes et al., 2008). Our study observed that treatment with MMB-2201 increased the levels of phospho-Ser9-GSK-3β upon activation of cannabinoid receptors. This finding indicates that GSK-3β is involved in intracellular signaling pathways activated by cannabinoid receptors that may contribute to promoting brain angiogenesis. This study demonstrates that direct exposure to MMB-2201 enhances the proliferation, migration, and angiogenic capability of HBEC-5i. MMB-2201 treatment resulted in elevated VEGF, ANG-1, and ANG-2 expression levels, highlighting their role in promoting angiogenesis. These findings underscore the association between these proteins and the angiogenic process. However, further investigations are needed to fully understand the mechanisms by which cannabinoids modulate VEGF expression and their therapeutic implications for cancer and other angiogenesis-related conditions. The study presents several potential limitations, including the off-target effects of MMB-2201, the challenge of generalizing in vitro findings to in vivo systems, and the long-term implications of synthetic cannabinoid use. Moreover, future research should prioritize investigating various angiogenesis-related factors, such as Insulin-like Growth Factors (IGFs) and Tumor Necrosis Factor (TNF), to elucidate how MMB-2201 exposure may influence activity through extensive gene and protein analysis. Additionally, it is crucial to identify other proteins associated with GSK-3β, including Akt and β-catenin, to gain a more comprehensive understanding of the underlying molecular mechanisms.

## Conclusion

This study demonstrates a notable increase in brain-associated angiogenesis, evidenced by the enhanced proliferation, migration, and angiogenic capability of HBEC5i cells in vitro following direct exposure to MMB-2201. This is the first study to explore the physiological effects of MMB-2201, shedding light on its potential therapeutic implications. Promoting angiogenesis is beneficial in conditions such as myocardial ischemia, where enhanced vascular growth can improve tissue repair and recovery. Conversely, in diseases like cancer, where excessive angiogenesis contributes to tumor growth, inhibition of angiogenesis is critical. Our findings underscore the potential of cannabinoid receptor modulators, including antagonists and agonists, as pharmacotherapeutics for managing angiogenesis-related diseases. However, several limitations should be noted. The challenge is translating these in vitro findings into clinical settings. The controlled conditions of in vitro studies may not fully reflect the complexity of human physiology, and the effects observed in HBEC-5i may differ in vivo due to numerous biological variables. Therefore, the effects observed in vitro must be validated in vivo to understand their therapeutic potential and safety profile fully.

Further investigation is required to explore the precise mechanisms through which MMB-2201 influences angiogenesis and to assess its efficacy and safety in animal models. Furthermore, no clinical trials on MMB-2201 regarding angiogenesis or related diseases have been conducted. Therefore, future research should include preclinical and clinical studies to confirm these findings and evaluate the clinical relevance of MMB-2201 as a therapeutic agent. It is necessary to address these challenges to translate these initial findings into safe and effective clinical therapeutics.

## Electronic supplementary material

Below is the link to the electronic supplementary material.

Supplementary Material 1

Supplementary Material 2

Supplementary Material 3

Supplementary Material 4

Supplementary Material 5

## Data Availability

All data generated or analyzed during this study are included in this published article.

## References

[CR1] Adair TH, Montani JP. (2010). Integrated Systems Physiology: from Molecule to Function to Disease. In *Angiogenesis*. Morgan & Claypool Life SciencesCopyright © 2010 by Morgan & Claypool Life Sciences. 10.4199/c00017ed1v01y201009isp009 21452444

[CR2] AL-Eitan L, Abu Kharmah H. The Effect of the synthetic cannabinoid AB-CHMINACA on the roles of vascular endothelial growth factor, Angiopoietin-1, and Angiopoietin-2 in Brain Angiogenesis. Appl Vitro Toxicol. 2023;9(3):104–15. 10.1089/aivt.2023.0003.

[CR4] Al-Eitan L, Abusirdaneh R. The synthetic cannabinoid 5-fluoro ABICA upregulates angiogenic markers and stimulates tube formation in human brain microvascular endothelial cells. J Taibah Univ Med Sci. 2024;19(2):359–71. 10.1016/j.jtumed.2024.01.002. 38357583 10.1016/j.jtumed.2024.01.002 PMC10864802

[CR5] Al-Eitan L, Alahmad S. The expression analyses of GSK3B, VEGF, ANG1, and ANG2 in human brain microvascular endothelial cells treated with the synthetic cannabinoid XLR-11. Gene. 2023;878:147585. 10.1016/j.gene.2023.147585. 37355149 10.1016/j.gene.2023.147585

[CR7] Al-Eitan L, Alkhawaldeh M. MDMB-FUBINACA influences Brain Angiogenesis and the expression of VEGF, ANG-1, and ANG-2. Curr Vasc Pharmacol. 2023;21(5):356–65. 10.2174/1570161121666230913093441. 37711102 10.2174/1570161121666230913093441

[CR3] Al-Eitan L, Kharmah HA. Effect of EMB-FUBINACA on brain endothelial cell angiogenesis: expression analysis of angiogenic markers. Naunyn Schmiedebergs Arch Pharmacol. 2024. 10.1007/s00210-024-03322-1. 39136736 10.1007/s00210-024-03322-1

[CR6] Al-Eitan L, Alhusban A, Alahmad S. Effects of the synthetic cannabinoid XLR-11 on the viability and migration rates of human brain microvascular endothelial cells in a clinically-relevant model. Pharmacol Rep. 2020;72(6):1717–24. 10.1007/s43440-020-00123-0. 32632915 10.1007/s43440-020-00123-0

[CR8] AL-Eitan LN, Alahmad SZ, ElMotasem MFM, Alghamdi MA. The synthetic cannabinoid 5F-MDMB-PICA enhances the metabolic activity and angiogenesis in human brain microvascular endothelial cells by upregulation of VEGF, ANG-1, and ANG-2. Toxicol Res. 2023. 10.1093/toxres/tfad068. 10.1093/toxres/tfad068 PMC10615825 37915478

[CR9] Al-Eitan LN, Alahmad SZ, ElMotasem MFM, Alghamdi MA. The synthetic cannabinoid 5F-MDMB-PICA enhances the metabolic activity and angiogenesis in human brain microvascular endothelial cells by upregulation of VEGF, ANG-1, and ANG-2. Toxicol Res (Camb). 2023;12(5):796–806. 10.1093/toxres/tfad068. 37915478 10.1093/toxres/tfad068 PMC10615825

[CR10] Al-Eitan LN, Zuhair S, Khair IY, Alghamdi MA. Assessment of the proliferative and angiogenic effects of the synthetic cannabinoid (R)-5-fluoro ADB on human cerebral microvascular endothelial cells. Iran J Basic Med Sci. 2024;27(3):304–10. 10.22038/ijbms.2023.71819.15605. 38333752 10.22038/IJBMS.2023.71819.15605 PMC10849210

[CR11] Annex BH, Cooke JP. New directions in therapeutic angiogenesis and arteriogenesis in peripheral arterial disease. Circul Res. 2021;128(12):1944–57. 10.1161/CIRCRESAHA.121.318266 PMC8538391 34110899

[CR12] Bai F, Guo F, Jiang T, Wei H, Zhou H, Yin H, Zhong H, Xiong L, Wang Q. Arachidonyl-2-Chloroethylamide alleviates cerebral ischemia Injury through glycogen synthase Kinase-3β-Mediated mitochondrial Biogenesis and Functional Improvement. Mol Neurobiol. 2017;54(2):1240–53. 10.1007/s12035-016-9731-7. 26820679 10.1007/s12035-016-9731-7

[CR13] Barceló B, Pichini S, López-Corominas V, Gomila I, Yates C, Busardò FP, Pellegrini M. Acute intoxication caused by synthetic cannabinoids 5F-ADB and MMB-2201: a case series. Forensic Sci Int. 2017;273:e10–4. 10.1016/j.forsciint.2017.01.020. 28190538 10.1016/j.forsciint.2017.01.020

[CR14] Barr JL, Unterwald EM. Glycogen synthase kinase-3 signaling in cellular and behavioral responses to psychostimulant drugs. Biochim Biophys Acta Mol Cell Res. 2020;1867(9):118746. 10.1016/j.bbamcr.2020.118746. 32454064 10.1016/j.bbamcr.2020.118746 PMC7313643

[CR15] Battaglin F, Puccini A, Intini R, Schirripa M, Ferro A, Bergamo F, Loupakis F. The role of tumor angiogenesis as a therapeutic target in colorectal cancer. Expert Rev Anticancer Ther. 2018;18(3):251–66. 29338550 10.1080/14737140.2018.1428092 PMC7493706

[CR16] Benedicto A, Arteta B, Duranti A, Alonso-Alconada D. The synthetic cannabinoid URB447 exerts Antitumor and Antimetastatic Effect in Melanoma and Colon cancer. Pharmaceuticals (Basel). 2022;15(10). 10.3390/ph15101166. 10.3390/ph15101166 PMC9606960 36297277

[CR17] Blázquez C, Casanova ML, Planas A, Del Gómez T, Villanueva C, Fernández-Aceñero MJ, Aragonés J, Huffman JW, Jorcano JL, Guzmán M. Inhibition of tumor angiogenesis by cannabinoids. Faseb J. 2003;17(3):529–31. 10.1096/fj.02-0795fje 12514108 10.1096/fj.02-0795fje

[CR18] Bogdanović V, Mrdjanović J, Borišev I. A review of the Therapeutic Antitumor potential of cannabinoids. J Altern Complement Med. 2017;23(11):831–6. 10.1089/acm.2017.0016. 28799775 10.1089/acm.2017.0016

[CR19] Brindle NP, Saharinen P, Alitalo K. Signaling and functions of angiopoietin-1 in vascular protection. Circ Res. 2006;98(8):1014–23. 10.1161/01.Res.0000218275.54089.12. 16645151 10.1161/01.RES.0000218275.54089.12 PMC2270395

[CR20] Carmeliet P. Angiogenesis in life, disease and medicine. Nature. 2005;438(7070):932–6. 16355210 10.1038/nature04478

[CR23] Carmeliet P, Jain RK. Molecular mechanisms and clinical applications of angiogenesis. Nature. 2011;473(7347):298–307. 10.1038/nature10144. 21593862 10.1038/nature10144 PMC4049445

[CR21] Carpi S, Fogli S, Polini B, Montagnani V, Podestà A, Breschi MC, Nieri P. Tumor-promoting effects of cannabinoid receptor type 1 in human melanoma cells. Toxicol Vitro. 2017;40:272–9. 10.1016/j.tiv.2017.01.018. 10.1016/j.tiv.2017.01.018 28131817

[CR22] Casanova ML, et al. Inhibition of skin tumor growth and angiogenesis in vivo by activation of cannabinoid receptors. J Clin Invest. 2003;111(1):43–50. 12511587 10.1172/JCI16116 PMC151833

[CR24] Cerretani D, Collodel G, Brizzi A, Fiaschi AI, Menchiari A, Moretti E, Moltoni L, Micheli L. Cytotoxic effects of cannabinoids on human HT-29 colorectal adenocarcinoma cells: different mechanisms of THC, CBD, and CB83. Int J Mol Sci. 2020;21(15). 10.3390/ijms21155533. 10.3390/ijms21155533 PMC7432098 32752303

[CR25] Cohen K, Weinstein A. The effects of cannabinoids on executive functions: evidence from Cannabis and Synthetic Cannabinoids-A systematic review. Brain Sci. 2018;8(3). 10.3390/brainsci8030040. 10.3390/brainsci8030040 PMC5870358 29495540

[CR26] Costas-Insua C, Seijo-Vila M, Blázquez C, Blasco-Benito S, Rodríguez-Baena FJ, Marsicano G, Guzmán M. Neuronal cannabinoid CB(1) receptors suppress the growth of Melanoma Brain metastases by inhibiting glutamatergic signalling. Cancers (Basel). 2023;15(9). 10.3390/cancers15092439. 10.3390/cancers15092439 PMC10177062 37173906

[CR27] Dillon M, Lopez A, Lin E, Sales D, Perets R, Jain P. Progress on Ras/MAPK Signaling Research and Targeting in blood and solid cancers. Cancers (Basel). 2021;13(20). 10.3390/cancers13205059. 10.3390/cancers13205059 PMC8534156 34680208

[CR28] Dudley AC, Griffioen AW. Pathological angiogenesis: mechanisms and therapeutic strategies. Angiogenesis. 2023;26(3):313–47. 10.1007/s10456-023-09876-7. 37060495 10.1007/s10456-023-09876-7 PMC10105163

[CR29] Faiz MB, Naeem F, Irfan M, Aslam MA, Estevinho LM, Ateşşahin DA, Sharifi-Rad J. Exploring the therapeutic potential of cannabinoids in cancer by modulating signaling pathways and addressing clinical challenges. Discover Oncol. 2024;15(1):490. 10.1007/s12672-024-01356-8 PMC11436528 39331301

[CR30] Ferrara N, Kerbel RS. Angiogenesis as a therapeutic target. Nature. 2005;438(7070):967. 16355214 10.1038/nature04483

[CR31] Flygare J, et al. Cannabinoid receptor ligands mediate growth inhibition and cell death in mantle cell lymphoma. FEBS Lett. 2005;579(30):6885–9. 16337199 10.1016/j.febslet.2005.11.020

[CR32] Gaunitz F, Andresen-Streichert H. Analytical findings in a non-fatal intoxication with the synthetic cannabinoid 5F-ADB (5F-MDMB-PINACA): a case report. Int J Legal Med. 2022;136(2):577–89. 34921326 10.1007/s00414-021-02717-6 PMC8847293

[CR33] Gholizadeh F, Ghahremani MH, Aliebrahimi S, Shadboorestan A, Ostad SN. Assessment of cannabinoids Agonist and Antagonist in Invasion potential of K562 Cancer cells. Iran Biomed J. 2019;23(2):153–8. 10.29252/.23.2.153. 29883990 10.29252/.23.2.153 PMC6707105

[CR34] Glogauer J, Blay J. Cannabinoids, their cellular receptors, and effects on the invasive phenotype of carcinoma and metastasis. Cancer Rep (Hoboken). 2022;5(2):e1475. 10.1002/cnr2.1475. 34313032 10.1002/cnr2.1475 PMC8842690

[CR35] Grimaldi C, et al. Anandamide inhibits adhesion and migration of breast cancer cells. Exp Cell Res. 2006;312(4):363–73. 16343481 10.1016/j.yexcr.2005.10.024

[CR36] Hinz B, Ramer R. Cannabinoids as anticancer drugs: current status of preclinical research. Br J Cancer. 2022;127(1):1–13. 10.1038/s41416-022-01727-4. 35277658 10.1038/s41416-022-01727-4 PMC9276677

[CR37] Hohmann T, Feese K, Greither T, Ghadban C, Jäger V, Dehghani F, Grabiec U. Synthetic Cannabinoids Influence the Invasion of Glioblastoma Cell Lines in a Cell- and Receptor-Dependent Manner. Cancers (Basel). 2019;11(2). 10.3390/cancers11020161 10.3390/cancers11020161 PMC6406558 30709059

[CR82] Holmes, T., O’Brien, T. A., Knight, R., Lindeman, R., Symonds, G., & Dolnikov, A. The role of glycogen synthase kinase-3beta in normal haematopoiesis, angiogenesis and leukaemia. Curr Med Chem. 2008;15(15):1493–1499. 10.2174/092986708784638834. 10.2174/092986708784638834 18537625

[CR38] Howlett AC, Blume LC, Dalton GD. CB(1) cannabinoid receptors and their associated proteins. Curr Med Chem. 2010;17(14):1382–93. 10.2174/092986710790980023. 20166926 10.2174/092986710790980023 PMC3179980

[CR39] Hur EM, Zhou FQ. GSK3 signalling in neural development. Nat Rev Neurosci. 2010;11(8):539–51. 10.1038/nrn2870. 20648061 10.1038/nrn2870 PMC3533361

[CR40] Jaworski T, Banach-Kasper E, Gralec K. GSK-3β at the intersection of neuronal plasticity and neurodegeneration. Neural Plast. 2019;2019:4209475. 10.1155/2019/4209475. 31191636 10.1155/2019/4209475 PMC6525914

[CR41] Jiang W, Zhang Y, Xiao L, Van Cleemput J, Ji SP, Bai G, Zhang X. Cannabinoids promote embryonic and adult hippocampus neurogenesis and produce anxiolytic- and antidepressant-like effects. J Clin Invest. 2005;115(11):3104–16. 10.1172/jci25509. 16224541 10.1172/JCI25509 PMC1253627

[CR42] Justinová Z, Yasar S, Redhi GH, Goldberg SR. The endogenous cannabinoid 2-arachidonoylglycerol is intravenously self-administered by squirrel monkeys. J Neurosci. 2011;31(19):7043–8. 10.1523/jneurosci.6058-10.2011. 21562266 10.1523/JNEUROSCI.6058-10.2011 PMC3123903

[CR43] Justus CR, Leffler N, Ruiz-Echevarria M, Yang LV. In vitro cell migration and invasion assays. J Vis Exp. 2014;88 10.3791/51046. PMC4186330 24962652

[CR44] Kerr AL, Steuer EL, Pochtarev V, Swain RA. Angiogenesis but not neurogenesis is critical for normal learning and memory acquisition. Neuroscience. 2010;171(1):214–26. 10.1016/j.neuroscience.2010.08.008. 20804819 10.1016/j.neuroscience.2010.08.008

[CR45] Kim HS, Skurk C, Thomas SR, Bialik A, Suhara T, Kureishi Y, Birnbaum M, Keaney JF Jr., Walsh K. Regulation of angiogenesis by glycogen synthase kinase-3beta. J Biol Chem. 2002;277(44):41888–96. 10.1074/jbc.M206657200. 12167628 10.1074/jbc.M206657200

[CR46] Kobayashi T, Hino S, Oue N, Asahara T, Zollo M, Yasui W, Kikuchi A. Glycogen synthase kinase 3 and h-prune regulate cell migration by modulating focal adhesions. Mol Cell Biol. 2006;26(3):898–911. 10.1128/MCB.26.3.898-911.2006. 16428445 10.1128/MCB.26.3.898-911.2006 PMC1347031

[CR47] Kokona D, Georgiou PC, Kounenidakis M, Kiagiadaki F, Thermos K. (2016). Endogenous and Synthetic Cannabinoids as Therapeutics in Retinal Disease. *Neural Plast*, *2016*, 8373020. 10.1155/2016/8373020 10.1155/2016/8373020 PMC4736800 26881135

[CR48] Le Boisselier R, Alexandre J, Lelong-Boulouard V, Debruyne D. Focus on cannabinoids and synthetic cannabinoids. Clin Pharmacol Ther. 2017;101(2):220–9. 10.1002/cpt.563. 27861784 10.1002/cpt.563

[CR49] Lee HS, Han J, Bai HJ, Kim KW. Brain angiogenesis in developmental and pathological processes: regulation, molecular and cellular communication at the neurovascular interface. Febs j. 2009;276(17):4622–35. 10.1111/j.1742-4658.2009.07174.x. 19664072 10.1111/j.1742-4658.2009.07174.x

[CR50] Lingegowda H, Miller JE, Marks RM, Symons LK, Alward T, Lomax AE, Koti M, Tayade C. Synthetic cannabinoid agonist WIN 55212-2 targets proliferation, angiogenesis, and apoptosis via MAPK/AKT Signaling in Human Endometriotic Cell Lines and a murine model of endometriosis. Front Reprod Health. 2021;3:726936. 10.3389/frph.2021.726936. 36304004 10.3389/frph.2021.726936 PMC9580784

[CR51] Luo H, et al. Cannabidiol increases Proliferation, Migration, Tubulogenesis, and Integrity of Human Brain endothelial cells through TRPV2 activation. Mol Pharm. 2019;16(3):1312–26. 30721081 10.1021/acs.molpharmaceut.8b01252

[CR52] Mackie K. Cannabinoid receptors: where they are and what they do. J Neuroendocrinol. 2008;20(1):10–4. 10.1111/j.1365-2826.2008.01671.x. 18426493 10.1111/j.1365-2826.2008.01671.x

[CR53] Marosi M, Arman P, Aceto G, D’Ascenzo M, Laezza F. Glycogen synthase kinase 3: Ion channels, plasticity, and diseases. Int J Mol Sci. 2022;23(8). 10.3390/ijms23084413. 10.3390/ijms23084413 PMC9028019 35457230

[CR55] Martínez-Martínez E, Gómez I, Martín P, Sánchez A, Román L, Tejerina E, García JM. Cannabinoids receptor type 2, CB2, expression correlates with human colon cancer progression and predicts patient survival. Oncoscience. 2015;2(2):131–41. 10.18632/oncoscience.119. 25859556 10.18632/oncoscience.119 PMC4381706

[CR54] Melincovici CS, Boşca AB, Şuşman S, Mărginean M, Mihu C, Istrate M, Moldovan IM, Roman AL, Mihu CM. Vascular endothelial growth factor (VEGF) - key factor in normal and pathological angiogenesis. Rom J Morphol Embryol. 2018;59(2):455–67. 30173249

[CR57] Moritz F, Schniering J, Distler JHW, Gay RE, Gay S, Distler O, Maurer B. Tie2 as a novel key factor of microangiopathy in systemic sclerosis. Arthritis Res Ther. 2017;19(1):105. 10.1186/s13075-017-1304-2. 28545512 10.1186/s13075-017-1304-2 PMC5445339

[CR56] Moriya J, Minamino T. Angiogenesis, cancer, and vascular aging. Front Cardiovasc Med. 2017;4:65. 29114540 10.3389/fcvm.2017.00065 PMC5660731

[CR58] Palmer TD, Willhoite AR, Gage FH. Vascular niche for adult hippocampal neurogenesis. J Comp Neurol. 2000;425(4):479–94. 10.1002/1096-9861(20001002)425:4%3C479::aid-cne2%3E3.0.co;2-3. 10975875

[CR59] Pisanti S, Bifulco M. Endocannabinoid system modulation in cancer biology and therapy. Pharmacol Res. 2009;60(2):107–16. 10.1016/j.phrs.2009.03.011. 19559362 10.1016/j.phrs.2009.03.011

[CR60] Pisanti S, Picardi P, Prota L, Proto MC, Laezza C, McGuire PG, Morbidelli L, Gazzerro P, Ziche M, Das A, Bifulco M. Genetic and pharmacologic inactivation of cannabinoid CB1 receptor inhibits angiogenesis. Blood. 2011;117(20):5541–50. 10.1182/blood-2010-09-307355. 21460248 10.1182/blood-2010-09-307355

[CR62] Portella G, et al. Inhibitory effects of cannabinoid CB1 receptor stimulation on tumor growth and metastatic spreading: actions on signals involved in angiogenesis and metastasis. Faseb J. 2003;17(12):1771–3. 12958205 10.1096/fj.02-1129fje

[CR63] Prateeksha P, Sharma VK, Singh SM, Sharma M, Diwan D, Hesham AE-L, Singh BN. Tetrahydrocannabinols: potential cannabimimetic agents for cancer therapy. Cancer Metastasis Rev. 2023;42(3):823–45. 36696005 10.1007/s10555-023-10078-2

[CR64] Santoro A, et al. Rimonabant inhibits human colon cancer cell growth and reduces the formation of precancerous lesions in the mouse colon. Int J Cancer. 2009;125(5):996–1003. 10.1002/ijc.24483 19479993

[CR641] Sarnataro D, et al. The cannabinoid CB1 receptor antagonist rimonabant (SR141716) inhibits human breast cancer cell proliferation through a lipid raft-mediated mechanism. Mol Pharmacol. 2006;70(4):1298–306. 10.1124/mol.106.025601 16822929

[CR65] Schurman LD, Lu D, Kendall DA, Howlett AC, Lichtman AH. Molecular mechanism and cannabinoid pharmacology. Handb Exp Pharmacol. 2020;258:323–53. 10.1007/164_2019_298. 32236882 10.1007/164_2019_298 PMC8637936

[CR66] Sezer Y, Jannuzzi AT, Huestis MA, Alpertunga B. In vitro assessment of the cytotoxic, genotoxic and oxidative stress effects of the synthetic cannabinoid JWH-018 in human SH-SY5Y neuronal cells. Toxicol Res (Camb). 2020;9(6):734–40. 10.1093/toxres/tfaa078. 33447358 10.1093/toxres/tfaa078 PMC7786167

[CR67] Sheik A, Farani MR, Kim E, Kim S, Gupta VK, Kumar K, Huh YS. Therapeutic targeting of the tumor microenvironments with cannabinoids and their analogs: update on clinical trials. Environ Res. 2023;231:115862. 37146933 10.1016/j.envres.2023.115862

[CR68] Shevyrin V, Melkozerov V, Nevero A, Eltsov O, Shafran Y, Morzherin Y, Lebedev AT. Identification and analytical characteristics of synthetic cannabinoids with an indazole-3-carboxamide structure bearing a N-1-methoxycarbonylalkyl group. Anal Bioanal Chem. 2015;407(21):6301–15. 10.1007/s00216-015-8612-7. 25893797 10.1007/s00216-015-8612-7

[CR69] Tai S, Fantegrossi WE. Synthetic cannabinoids: pharmacology, behavioral effects, and abuse potential. Curr Addict Rep. 2014;1(2):129–36. 10.1007/s40429-014-0014-y. 26413452 10.1007/s40429-014-0014-y PMC4582439

[CR70] Takahashi S. Vascular endothelial growth factor (VEGF), VEGF receptors and their inhibitors for antiangiogenic tumor therapy. Biol Pharm Bull. 2011;34(12):1785–8. 10.1248/bpb.34.1785. 22130231 10.1248/bpb.34.1785

[CR71] Trazzi S, Steger M, Mitrugno VM, Bartesaghi R, Ciani E. CB1 cannabinoid receptors increase neuronal precursor proliferation through AKT/glycogen synthase kinase-3beta/beta-catenin signaling. J Biol Chem. 2010;285(13):10098–109. 10.1074/jbc.M109.043711. 20083607 10.1074/jbc.M109.043711 PMC2843172

[CR72] Tuteja N. Signaling through G protein coupled receptors. Plant Signal Behav. 2009;4(10):942–7. 10.4161/psb.4.10.9530. 19826234 10.4161/psb.4.10.9530 PMC2801357

[CR73] Walsh KB, Andersen HK. Molecular Pharmacology of Synthetic cannabinoids: delineating CB1 receptor-mediated Cell Signaling. Int J Mol Sci. 2020;21(17). 10.3390/ijms21176115. 10.3390/ijms21176115 PMC7503917 32854313

[CR74] Wang FT, Sun W, Zhang JT, Fan YZ. Cancer–associated fibroblast regulation of tumor neo–angiogenesis as a therapeutic target in cancer. Oncol Lett. 2019;17(3):3055–65. 30867734 10.3892/ol.2019.9973 PMC6396119

[CR75] Xue C, Li X, Ba L, Zhang M, Yang Y, Gao Y, Sun Z, Han Q, Zhao RC. MSC-Derived exosomes can enhance the angiogenesis of human brain MECs and show therapeutic potential in a mouse model of Parkinson’s Disease. Aging Dis. 2021;12(5):1211–22. 10.14336/ad.2020.1221. 34341703 10.14336/AD.2020.1221 PMC8279521

[CR76] Yin S. Adolescents and drug abuse: 21st Century Synthetic substances. Clin Pediatr Emerg Med. 2019;20(1):17–24. 10.1016/j.cpem.2019.03.003.

[CR77] Yuan F, Wang Y, Guan Y, Ren Y, Lu H, Xiao T, Xie H, Vosler PS, Chen J, Yang GY. Real-time imaging of mouse lenticulostriate artery following brain ischemia. Front Biosci (Elite Ed). 2013;5(2):517–24. 10.2741/e633. 23277007 10.2741/e633

[CR78] Zhang ZG, Zhang L, Jiang Q, Zhang R, Davies K, Powers C, Bruggen N, Chopp M. VEGF enhances angiogenesis and promotes blood-brain barrier leakage in the ischemic brain. J Clin Invest. 2000;106(7):829–38. 10.1172/jci9369. 11018070 10.1172/JCI9369 PMC517814

[CR79] Zhang Y, Zheng W, Shen K, Shen W. ∆9-tetrahydrocannabinol inhibits epithelial-mesenchymal transition and metastasis by targeting matrix metalloproteinase-9 in endometrial cancer. Oncol Lett. 2018;15(6):8527–35. 10.3892/ol.2018.8407. 29805589 10.3892/ol.2018.8407 PMC5950514

[CR80] Zhao P, Li Q, Shi Z, Li C, Wang L, Liu X, Jiang C, Qian X, You Y, Liu N, Liu LZ, Ding L, Jiang BH. GSK-3β regulates tumor growth and angiogenesis in human glioma cells. Oncotarget. 2015;6(31):31901–15. 10.18632/oncotarget.5043. 26388612 10.18632/oncotarget.5043 PMC4741649

[CR81] Zou S, Kumar U. Cannabinoid receptors and the Endocannabinoid System: signaling and function in the Central Nervous System. Int J Mol Sci. 2018;19(3). 10.3390/ijms19030833. 10.3390/ijms19030833 PMC5877694 29533978

