# Supplementary material for: Evaluation of the metabolic activity, angiogenic impacts, and GSK-3β signaling of the synthetic cannabinoid MMB-2201 on human cerebral microvascular endothelial cells
Source: J Cannabis Res. 2024 Dec 20;6:43. doi: 10.1186/s42238-024-00255-7 (PMC11660800; doi:10.1186/s42238-024-00255-7)

**Cannabinoid Receptor Type 1**


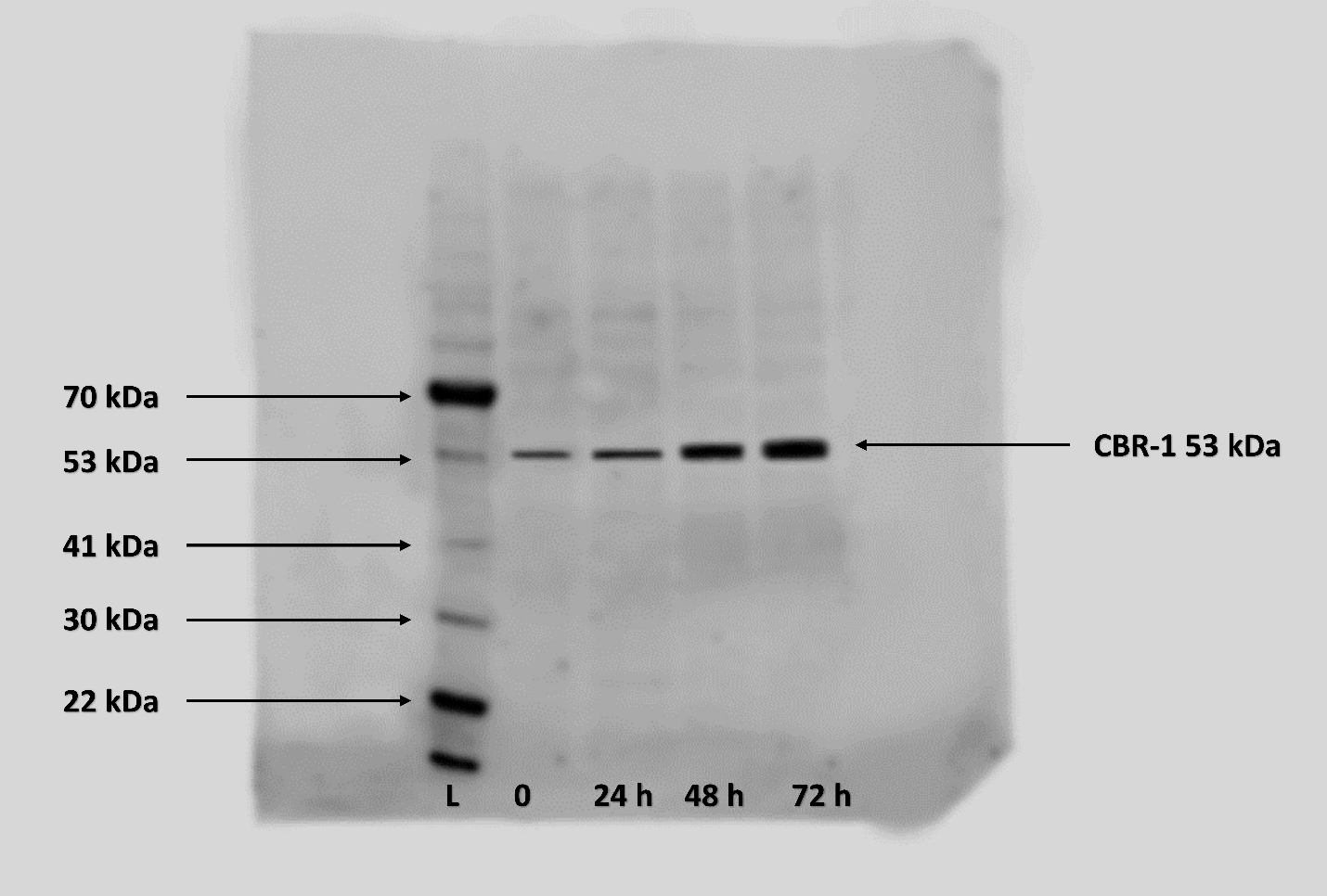


**β-Actin**


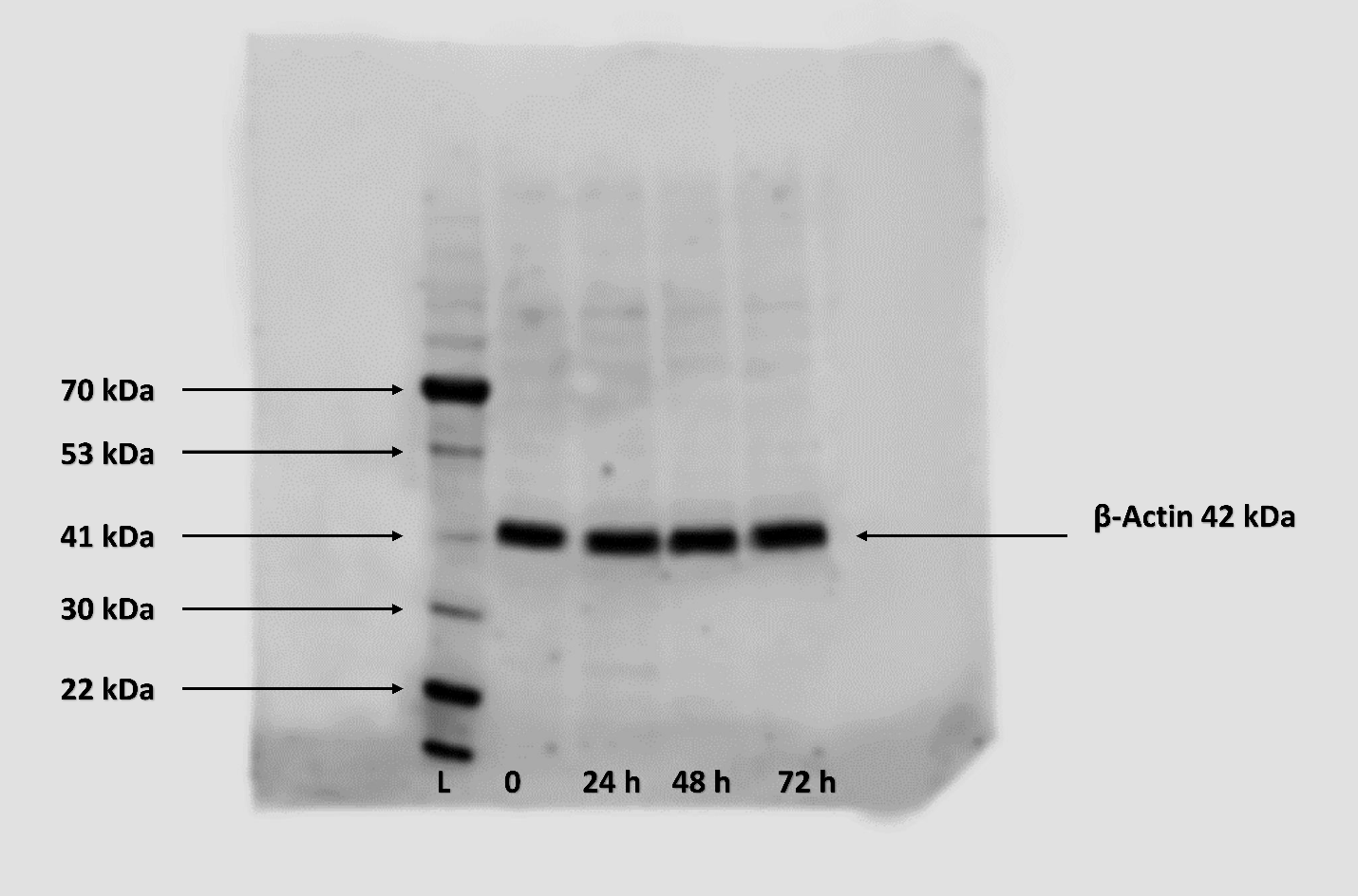


**Vascular Endothelial Growth Factor**


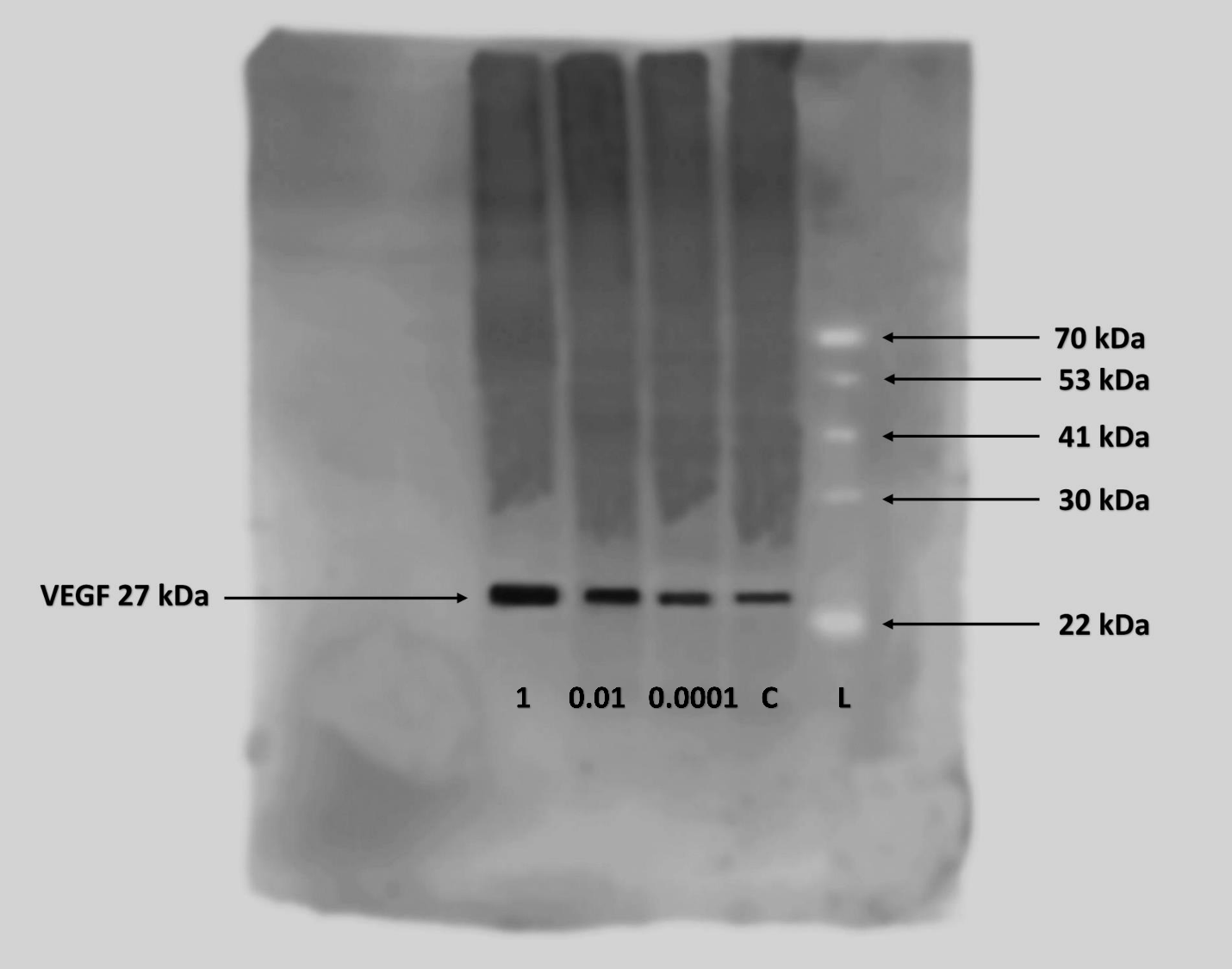


**Angiopoietin-1**


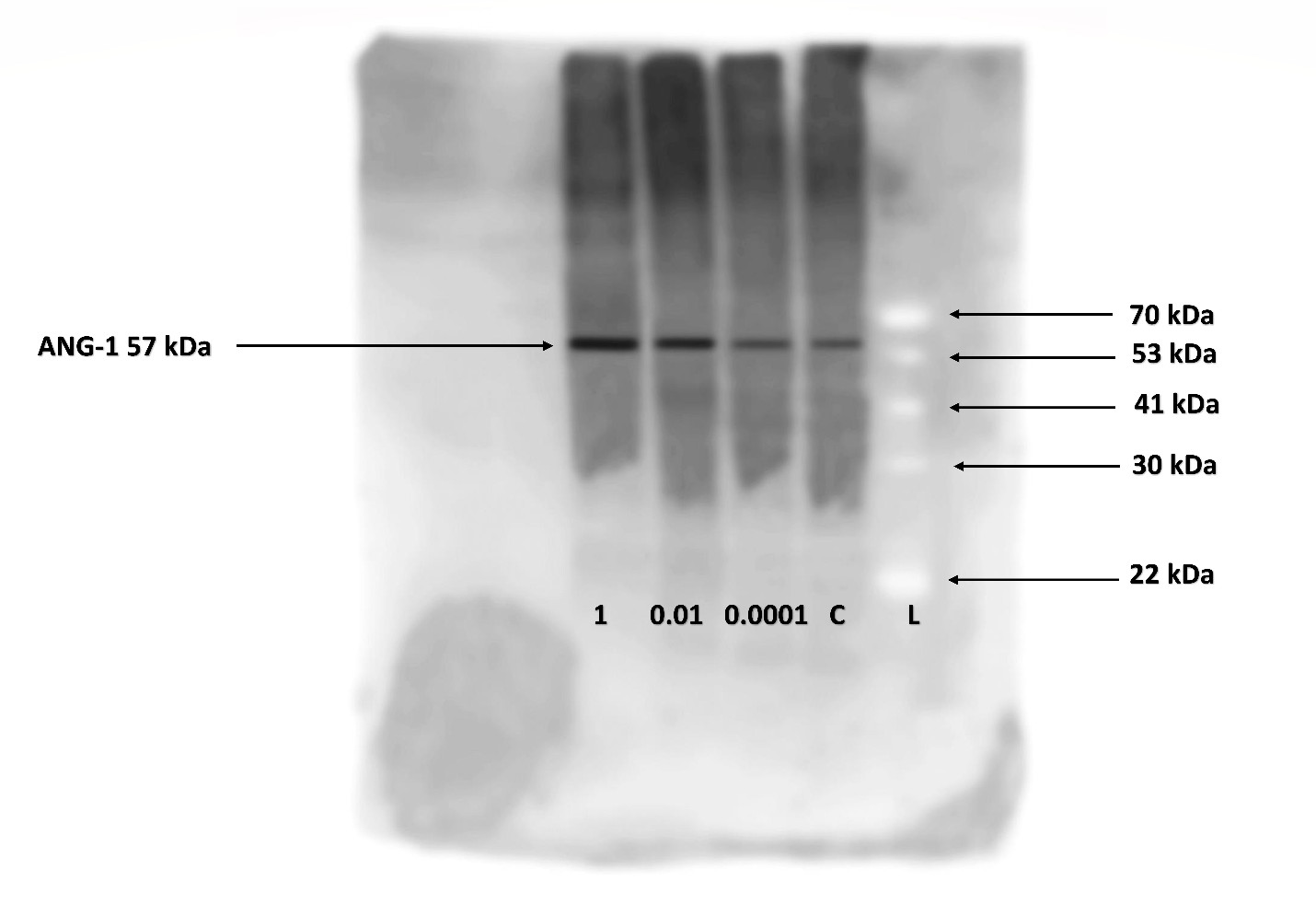


**β-Actin**


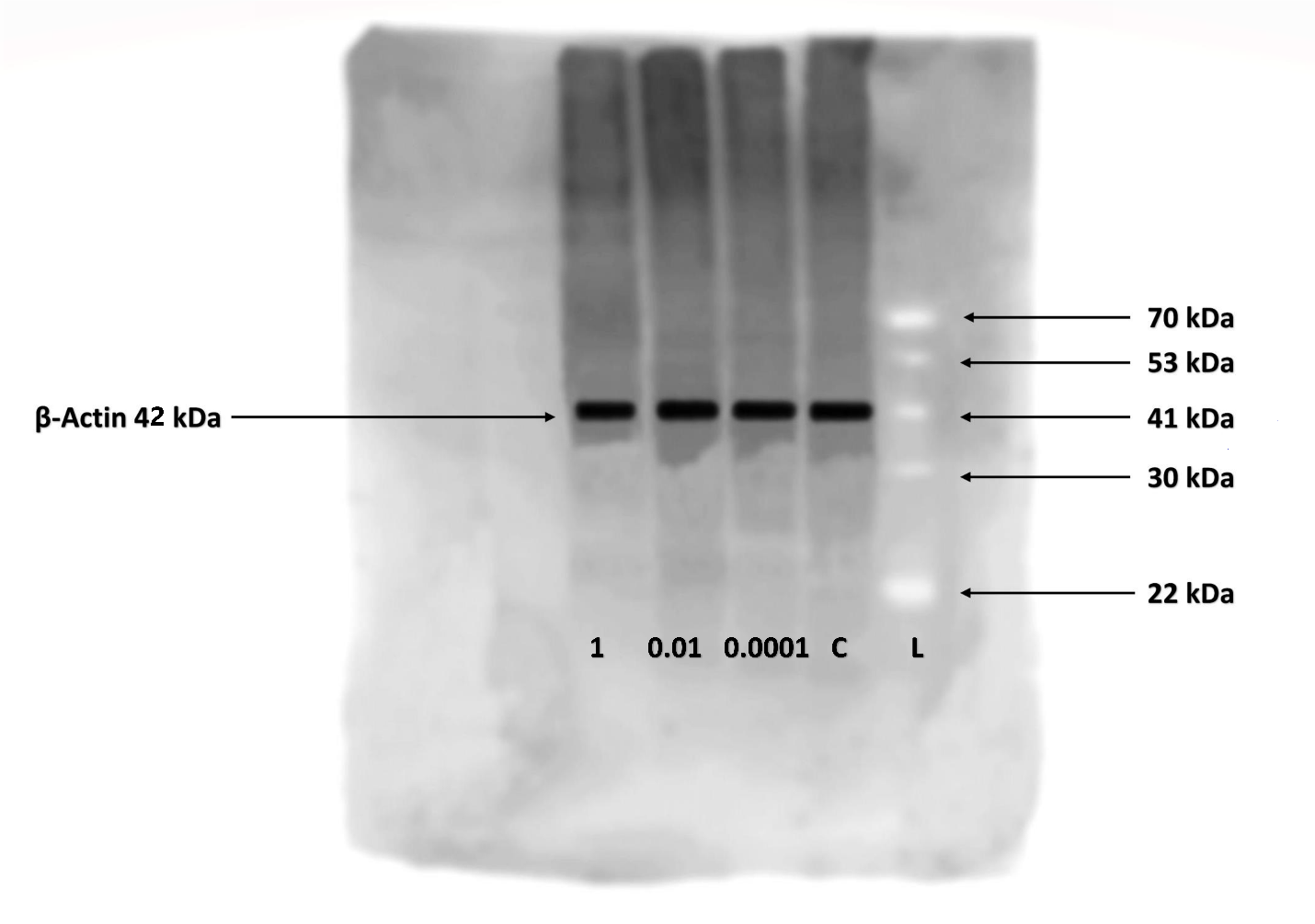


**Angiopoietin-2**


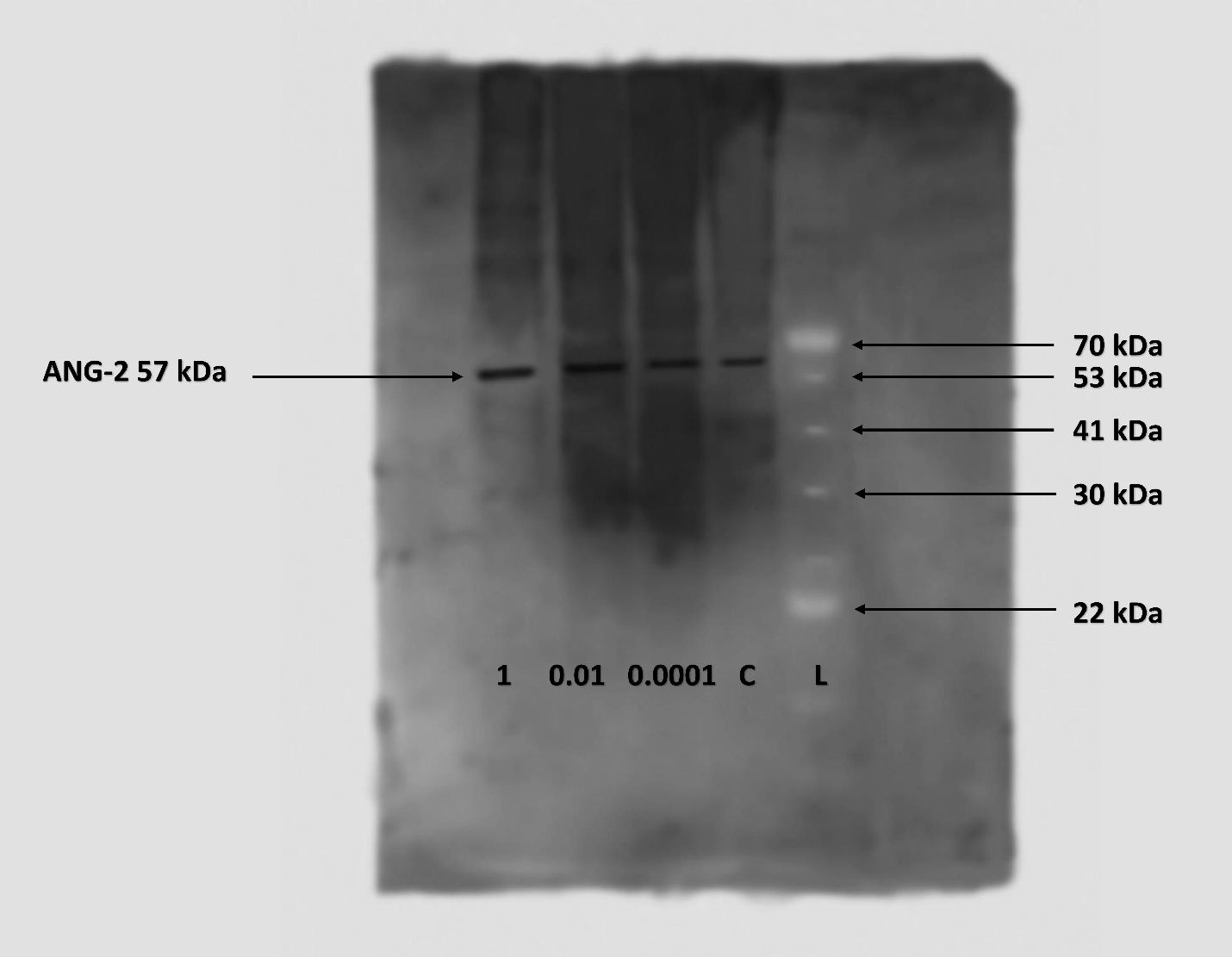


**β-Actin**


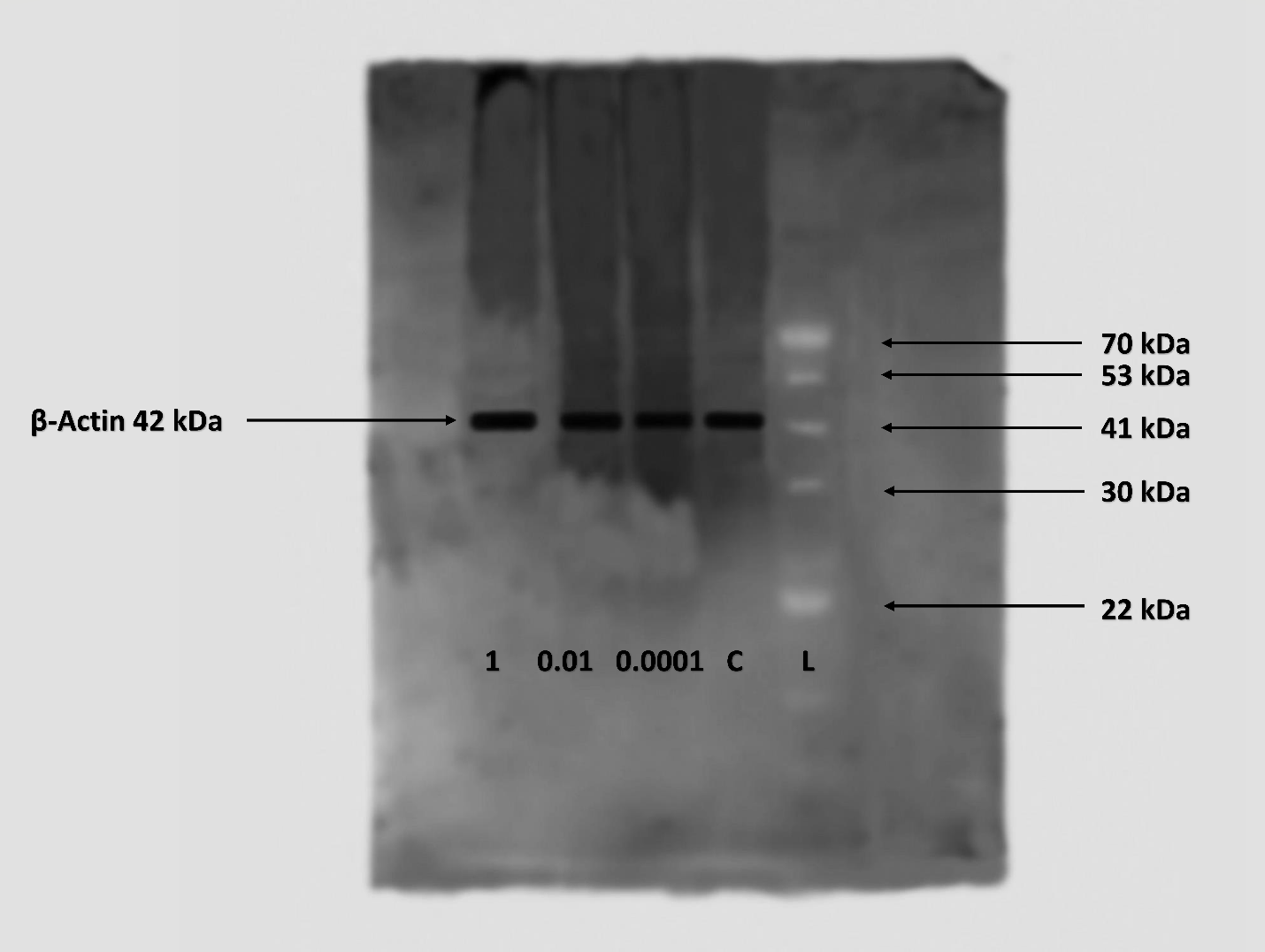


**Total Glycogen Synthase Kinase 3β**


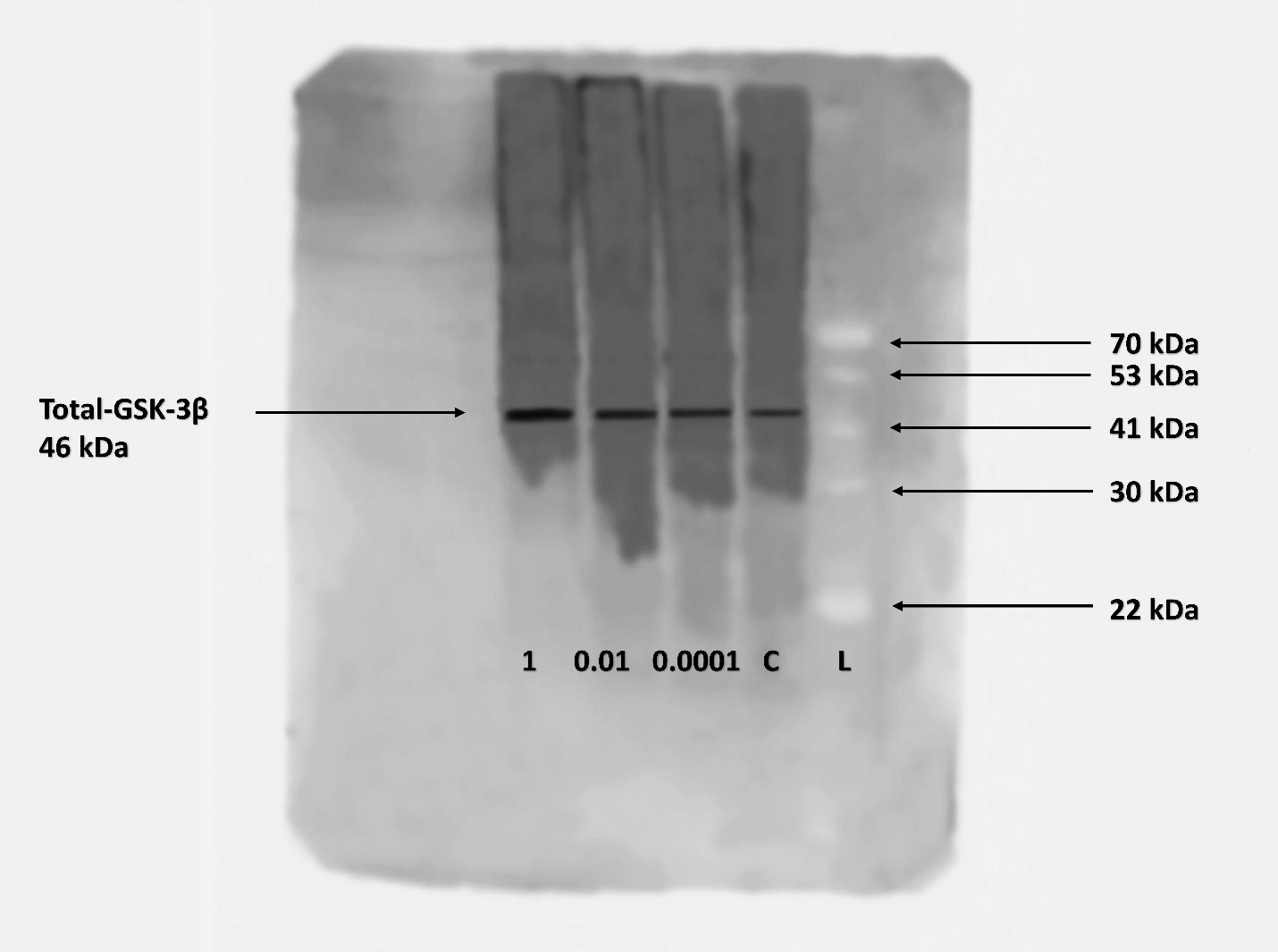


**Phosphorylated Ser9-Glycogen Synthase Kinase 3β**


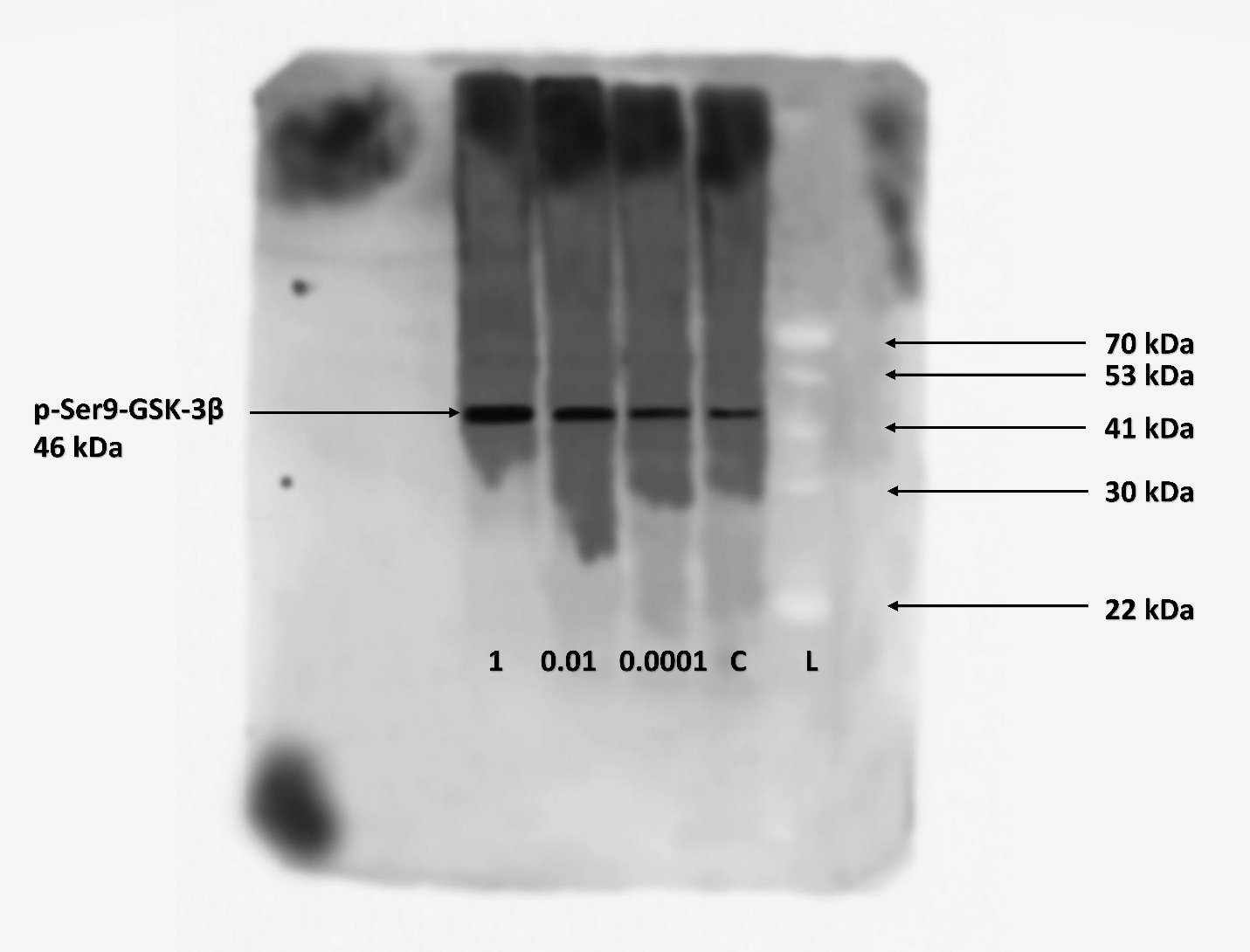

Supplement: Supplementary file 1 — Supplementary Material 1 [file 42238_2024_255_MOESM1_ESM.docx]
