# Supplementary material for: Evaluation of the metabolic activity, angiogenic impacts, and GSK-3β signaling of the synthetic cannabinoid MMB-2201 on human cerebral microvascular endothelial cells
Source: J Cannabis Res. 2024 Dec 20;6:43. doi: 10.1186/s42238-024-00255-7 (PMC11660800; doi:10.1186/s42238-024-00255-7)
Supplement: Supplementary file 3 — Supplementary Material 3 [file 42238_2024_255_MOESM3_ESM.docx]

**Supplementary Table 1: List of antibodies used in the Immunoblotting assay.**

| Factor | Primary Antibodies | Company |
| --- | --- | --- |
| VEGF | Anti-VEGF (Ab46154) | Abcam |
| ANG-1 | Anti-ANG-1 (Ab94684) | Abcam |
| ANG-2 | Anti-ANG-2 (Ab15393) | Abcam |
| Cannabinoid Receptor type 1 | Anti-Cannabinoid Receptor Type 1 (Ab259323) | Abcam |
| Total-GSK3β | Anti-Total-GSK3β (PA5-95845) | ThermoFisher |
| Phospho-Ser9-GSK3 β | Anti-Phospho-Ser9-GSK3 Β (9336S) | Cell Signaling Technology |
| Anti-β-Actin | Anti-β-Actin (4967S) | Cell Signaling Technology |
