# Supplementary material for: Evaluation of the metabolic activity, angiogenic impacts, and GSK-3β signaling of the synthetic cannabinoid MMB-2201 on human cerebral microvascular endothelial cells
Source: J Cannabis Res. 2024 Dec 20;6:43. doi: 10.1186/s42238-024-00255-7 (PMC11660800; doi:10.1186/s42238-024-00255-7)
Supplement: Supplementary file 4 — Supplementary Material 4 [file 42238_2024_255_MOESM4_ESM.docx]

**Supplementary Table 2.** RT-qPCR program used for amplification and quantification of RNA.

| **Step** | | **Protocol** |
| --- | --- | --- |
| Reverse transcription | | 50 °C, 15 min |
| Enzyme activation | | 95 °C, 10 min |
| 40 Cycles | Denaturation | 95 °C, 10 sec |
|  | Annealing | 60 °C, 20 sec |
|  | Extension | 72 °C, 20 sec |
