# Supplementary material for: Evaluation of the metabolic activity, angiogenic impacts, and GSK-3β signaling of the synthetic cannabinoid MMB-2201 on human cerebral microvascular endothelial cells
Source: J Cannabis Res. 2024 Dec 20;6:43. doi: 10.1186/s42238-024-00255-7 (PMC11660800; doi:10.1186/s42238-024-00255-7)
Supplement: Supplementary file 5 — Supplementary Material 5 [file 42238_2024_255_MOESM5_ESM.docx]

**Supplementary Table 2.** Primer sequences used in RT-qPCR.

| **Primer** | **Forward Sequence** | **Reverse Sequence** |
| --- | --- | --- |
| β-actin | 5′-GGAGATTACTGCCCTGGCTCCTA-3′ | 5′-GACTCATCGTACTCCTGCTTGCTG-3′ |
| VEGF | 5′-GCACGTTGGCTCACTTCCAG-3′ | 5′-TGGTCGGAACCAGAATCTTTATCTC-3′ |
| ANG-1 | 5′-ACCGTGAGGATGGAAGCCTAGA-3′ | 5′-AATGAACTCGTTCCCAAGCCAATA-3′ |
| ANG-2 | 5′-CTTCAAGTCAGGACTCACCACCA-3′ | 5′-CCACCCATGTCCATGTCACAG-3′ |
